# Association of Participation in an End-of-Life Conversation Game With Advance Care Planning Behavior and Perspectives Among African American Individuals

**DOI:** 10.1001/jamanetworkopen.2020.4315

**Published:** 2020-05-08

**Authors:** Lauren Jodi Van Scoy, Benjamin H. Levi, Pamela Witt, Cindy Bramble, Christopher Richardson, Irene Putzig, A. Rose Levi, Emily Wasserman, Vernon Chinchilli, Amy Tucci, Michael J. Green

**Affiliations:** 1 Department of Medicine, Penn State Milton S. Hershey Medical Center, Penn State College of Medicine, Hershey, Pennsylvania; 2 Department of Humanities, Penn State Milton S. Hershey Medical Center, Penn State College of Medicine, Hershey, Pennsylvania; 3 Department of Public Health Sciences, Penn State Milton S. Hershey Medical Center, Penn State College of Medicine, Hershey, Pennsylvania; 4 Department of Pediatrics, Penn State Milton S. Hershey Medical Center, Penn State College of Medicine, Hershey, Pennsylvania; 5 Hospice Foundation of America, Washington, DC

## Abstract

**Importance:**

Less than 25% of African American individuals have completed advance directives and are thus vulnerable to poor end-of-life care. Low-cost interventions are needed to increase engagement in advance care planning (ACP).

**Objectives:**

To investigate whether an end-of-life conversation game motivates African American attendees to engage in ACP and to assess whether the game is well received and endorsed.

**Exposures:**

Attendance at an end-of-life conversation game (*Hello)* played in groups of 4 to 6 participants for 60 minutes.

**Design, Setting, and Participants:**

Prospective, mixed-methods cohort study conducted from 2018 to 2019 with a 3- to 11-month follow-up interview. Game events were held in 53 community venues across the US; 15 were purposively sampled for onsite research procedures. Of 428 attendees at purposively sampled sites, 386 (90%) consented to research procedures (6 attendees were removed from analysis for protocol deviation). Of 367 attendees who provided accurate contact information, 232 (63%) were contacted, and 220 were included in follow-up analyses.

**Main Outcomes and Measures:**

The primary outcome was advance directive completion rates after the intervention. Secondary outcomes included rates of other ACP behaviors, ACP engagement, conversation satisfaction and realism, and participants’ Net Promoter Score (a measure of endorsement). Follow-up telephone interviews explored the game experience and relevant ACP behaviors of attendees.

**Results:**

Of 380 individuals who participated (mean [SD] age, 62.2 [13.8] years; 304 were female [80%], and 348 were [92%] African American), none withdrew because of an adverse event. After the intervention, 91 of 220 attendees (41%) completed a new advance directive; 176 of 220 attendees (80%) discussed end-of-life wishes with loved ones, and 214 of 219 attendees (98%) completed at least 1 ACP behavior. There was a moderate increase in the self-efficacy domain on the ACP Engagement Survey (mean [SD] change from before to after the game, 0.54 [0.98]; *P* < .001). The mean (SD) conversation satisfaction score was 6.21 (0.93) (range, 1-7, with 7 being highest satisfaction), and the overall Net Promoter Score was 57.89 (range, −100 to 100, with 100 being highest endorsement). Interviews revealed 5 themes about the game: (1) it was a useful forum for ACP; (2) it provided new information and perspective; (3) it was emotionally beneficial; (4) it increased appreciation for ACP; and (5) it empowered and motivated participants to perform ACP. Mixed-methods integration showed convergence across data sets.

**Conclusions and Relevance:**

Among a nationwide sample of African American individuals, the end-of-life conversation game appeared to be well received and was associated with high rates of ACP behavior. This low-cost and scalable tool may help reduce health disparities associated with end-of-life care.

## Key Points

**Question:**

Can a low-cost, easily scaled, end-of-life conversation game motivate underserved African American individuals to engage in advance care planning?

**Findings:**

This national mixed-methods cohort study reaching 384 underserved African American individuals found that high rates of advance care planning behavior were associated with participation in game events at community venues.

**Meaning:**

The end-of-life conversation game may be a useful tool for engaging underserved African American communities in advance care planning, a step toward reducing health disparities related to end-of-life care.

## Teaser

This national mixed-methods cohort study investigates whether an end-of-life conversation game is well received and endorsed and motivates advance care planning among underserved African American individuals.

## Introduction

Underserved populations, particularly African American communities, are vulnerable to low-quality end-of-life care.^1^ Compared with white individuals living in the United States, African American individuals are less likely to receive end-of-life care aligned with their preferences^2,3,4^ and are less likely to receive hospice services.^5,6^ Such disparities can be addressed in part by advance care planning (ACP)—a process involving conversations about values and preferences for end-of-life care, documentation in advance directives (ADs), and periodic reviews or updates.^7^ The completion of an AD is associated with reduced unwanted end-of-life medical interventions,^8,9,10^ increased hospice use,^11^ and decreased psychological distress^9,12^ and may reduce end-of-life costs.^13,14,15^

While the percentage of individuals in the US engaging in ACP has nearly doubled to approximately 60% in the last decade, among African American individuals, it remains stagnant at less than 25%.^1,16,17,18,19^ Most strategies for increasing ACP involve resource-intensive 1-to-1 encounters with clinicians, an approach not easily scaled.^20,21^ Furthermore, traditional approaches to ACP neglect the 2 most well-documented barriers among underserved populations: mistrust of the health care system^22,23,24^ and reluctance to discuss dying.^22,24^ Our team sought to address these issues by evaluating an inexpensive and easily disseminated intervention—a serious game that promotes ACP conversations by combining an important topic with an enjoyable activity to help overcome reluctance to discuss death and dying.^25,26,27^

In prior research, participants have reported that the game’s open-ended questions prompted in-depth discussions of values and preferences about end-of-life care,^25,26,27,28,29^ with 98% subsequently performing at least 1 ACP behavior (eg, AD completion or discussing end-of-life issues with loved ones).^25,26,28^ However, these studies were conducted primarily in white and South Asian communities. The present study examined the feasibility and acceptability of the game in underserved African American populations and explored whether the game empowered them to complete ACP.

To overcome barriers associated with skepticism about ACP and distrust of the health care system, we developed a pragmatic, community-based delivery model leveraging social networks. We hypothesized that the game would be highly endorsed and engaging for underserved African American communities.

## Methods

### Study Design

This was a nationally scaled, prospective, mixed-methods cohort study. The primary outcome was completion of a new AD or review or an update of an existing AD within 3 to 11 months after finishing the game. Although a randomized clinical trial may permit conclusions regarding causation, project organizers expressed preferences for giving all participating communities access to the intervention and thus adopted the present mixed-methods cohort design. This study follows the Strengthening the Reporting of Observational Studies in Epidemiology (STROBE) reporting guideline for cohort studies and COREQ guidelines for qualitative data and was registered at ClinicalTrials.gov (NCT03456921). The Penn State Hershey Institutional Review Board approved the protocol. Participants provided verbal informed consent, which fulfilled the consent criteria set forth by the institutional review board for this minimal risk study, after reviewing a Summary Explanation of Research. Participants received a $20 gift card for completing the study activities after finishing the game.

### Community-Based Delivery Model and Sampling

The Hello Project was a national initiative that engaged geographically diverse individuals from underserved communities. Partnering with the nonprofit Hospice Foundation of America, we used a community-based delivery model to host game events. Influential community organizations (eg, places of worship and community centers) were recruited as hosts if they had experience engaging underserved communities. Using press releases and email listservs, we interviewed 63 applicants via telephone; 53 were selected based on community connections and demographic considerations. All hosts represented underserved communities defined by the National Institutes of Health as “including black/African Americans, socioeconomically disadvantaged, and rural communities.”^30^ On the basis of funder priority and the desire to address unmet ACP needs in these communities, 17 sites in African American communities were purposively sampled for onsite research based on geographic region and hosts’ prior outreach success. Hosts underwent training on running game events and managing logistics (eg, inviting participants, arranging venues, and introducing the game). Two sites were unable to schedule events within the project timeline. Research staff traveled to 15 sites and obtained informed consent and collected data. Taking a conservative approach, we excluded data from nonpurposively sampled sites because potential procedural variations could not be rigorously ruled out.

### Data Management

Onsite data were collected via paper forms and entered into a secure, electronic REDCap database. Telephone interviews were audiorecorded, and responses were entered into REDCap.

### Setting and Host Sites

The full scope of the project involved 15 purposively sampled sites and 38 nonpurposively sampled sites in 27 states. In total, 1122 individuals participated from 4 US regions: the Northeastern (n = 8), Southern (n = 24), Midwestern (n = 11), and Western (n = 10) regions. Urban (n = 32) and rural (n = 21) sites were included. Recruitment occurred from May to November 2018; follow-up calls were completed by September 2019. Differences between purposively and nonpurposively sampled sites were procedural in nature because purposively sampled sites had an onsite research assistant (eAppenidx 1 in the Supplement). Only data and procedures from purposively sampled sites are reported hereafter.

### Participant Recruitment

Hosts advertised events using institutional review board–approved fliers and newsletters. Participants were research eligible if they attended a game event and self-reported being 18 years of age or older. Those who did not speak English or self-reported difficulties with hearing or speaking were excluded from the analysis but were invited to play the game absent research questionnaires.

### Event Procedures

Before the intervention, the onsite research team administered questionnaires on demographic characteristics, health status, experience with medical decision-making, and ACP engagement. Hosts opened the event with a scripted greeting providing background about ACP and explaining the event agenda and game rules (eTable 1 in the Supplement). The game was then played for 60 minutes in groups of 4 to 6 participants, using a booklet of 32 open-ended questions (published previously).^27,29,31^ A player read aloud a question (eg, “In order to provide you with the best care possible, what 3 nonmedical facts should your doctor know about you?”). Players wrote answers and took turns sharing with the group (or were allowed to pass). Answers typically prompted free-flowing conversation. Players controlled how long they shared, what they shared, and when they were ready to proceed to the next question. Players could give others a game chip to acknowledge a particularly thoughtful comment. To promote lighthearted competition, a “winner” was named at the end, with a pregame coin flip determining whether the “winner” would be the player with the most or fewest chips revealed at the end.

#### Postintervention Measures

Immediately after the game, participants completed a questionnaire assessing game conversation satisfaction (8-item mean score ranging from 1 to 7, with 7 representing the highest satisfaction).^32^ Participants also completed a 5-item, validated questionnaire measuring conversation realism (5-item mean score ranging from 1 to 7, with 7 indicating most realistic).^33^ Intervention endorsement was assessed with the Net Promoter Score (NPS), a validated measure used widely in marketing research to estimate product uptake and recommendation.^34,35^ The single-item question asked “How likely is it that you would recommend the game to friends or family?” on a scale from 1 (not likely at all) to 10 (extremely likely).

#### Follow-up Measures

Follow-up telephone calls were made 3 to 11 months after each game event to administer questionnaires and conduct an audiorecorded interview about perceptions of the game and relevant actions taken after participating in the game (eAppendix 2 in the Supplement). Participants were contacted starting at 3 months after the intervention. Telephone calls were made until participants were interviewed or declined to be interviewed or 10 calls had been made without contact or 11 months had elapsed since the event. Participants not reached after 10 attempts or 11 months were considered lost to follow-up. The primary outcome was self-reported completion of an AD (defined as any legal document that provides guidance on medical decision-making). Self-report was necessary because review of medical records for AD documentation was not feasible for a study of this scope. Participants who completed ADs prior to the event were asked if they had reviewed or updated their existing AD because periodic review is considered an essential ACP behavior.^36^ Secondary outcomes included (1) completion of other ACP behaviors (eg, discussing end-of-life wishes with loved ones or clinicians and reviewing ACP resources) and (2) change in score on the 34-item validated ACP Engagement Survey from before the intervention to 3 to 11 months after the intervention.^37^ The ACP Engagement Survey can detect change in response to ACP interventions.^37,38^ We defined a moderate, clinically meaningful increase to be a score change of 0.50 to 0.79.^38,39^

### Statistical Analysis

#### Sample Size

The sample size was chosen in collaboration with the sponsor to reach a diverse, national sample of underserved individuals in the US, with emphasis on African American communities. The target sample size was 50 communities (20-50 participants per site event, anticipating 10% attrition). Owing to resource and staff limitations, 15 of the 53 sites were purposively selected to deploy research staff onsite for data collection. Site enrollment was stratified according to urban or rural area and US region (Table 1).

**Table 1.** Purposively Sampled Sites and Host Demographic Characteristics

Site	Venue type	Region	Urban or rural	Attendees, No.	Consent rate, No. (%)
Lafayette, Louisiana	Place of worship	South	Rural	43	43 (100)
Sodus, New York	Place of worship	Northeast	Rural	22	22 (100)
Tuscaloosa, Alabama	University site	South	Rural	52	50 (96)
Atlanta, Georgia	Health center	South	Urban	35	35 (100)
Philadelphia, Pennsylvania	Place of worship	Northeast	Urban	17	16 (94)
Amarillo, Texas	Community center	Midwest	Urban	20	20 (100)
Washington, DC	Place of worship	South	Urban	48	45 (94)
Las Vegas, Nevada	Senior center	West	Urban	39	36 (92)
Lakeland, Florida	Health center	South	Urban	24	23 (96)
Milwaukee, Wisconsin	Place of worship	Midwest	Urban	31	18 (58)
Asheville, North Carolina	Place of worship	South	Rural	24	24 (100)
Chicago, Illinois	Place of worship	Midwest	Urban	32	13 (41)
Battle Creek, Michigan^a^	Place of worship	Midwest	Urban	6	6 (100)
Palo Alto, California	Place of worship	West	Urban	18	18 (100)
St Louis, Missouri	Senior center	Midwest	Urban	17	17 (100)
Total purposively samples sites				428	386 (90)

^a^ Site removed from analysis due to low attendance resulting in protocol deviation.

#### Data and Statistical Methods

The AD completion rates and completion of other ACP behaviors were calculated. The 34-item ACP Engagement Survey consists of 34 items measured on 5-point Likert scale, with an overall mean and 4 domain scores^37^: knowledge (2 items), contemplation (3 items), self-efficacy (12 items), and readiness (17 items). Scores have strong internal consistency, test-retest reliability, and construct validity and have shown the ability to detect change in ACP behavior.^37,40^ The Wilcoxon signed rank test was used to assess changes in ACP Engagement Survey scores as the difference between the time of the follow-up call and immediately prior to the event, analyzing only respondents with scores at both time points. The NPS uses a 10-point Likert scale and classifies detractors (1-6), passives (7-8), or promoters (9-10).^41^ The NPS is calculated by taking the difference between the percentage of promoters and detractors (scores range from −100 to 100). Positive scores greater than 0 indicate positive endorsement. Conversation satisfaction scores were calculated by averaging 8 items from a 7-point Likert scale.^32^ Conversation realism is a 5-item mean score on a 7-point Likert scale.^33^

For all calculated scores, missing items resulted in a missing composite score. Follow-up time was calculated as the number of days between the event and the telephone interview (eAppendix 3 in the Supplement). All analyses were conducted using SAS, version 9.4 (SAS Institute Inc), and 2-sided tests with α = .05 were considered statistically significant.

#### Qualitative Analysis

Thematic analysis was applied to transcribed interviews. Two analysts (L.J.V.S. and A.R.L.) independently reviewed 20% of responses and created categories from the data to form a preliminary codebook. Codes within each category were defined and used to analyze another 20% of responses via the constant comparison method.^42^ After responses were independently coded, conflicts were reconciled through discussion, and the codebook was finalized. Two analysts (L.J.V.S. and A.R.L.) coded the remaining data and then organized the codes into themes.

## Results

Table 1 gives host site locations, venues, demographic characteristics, and consent rates at each purposively sampled site. One site was excluded from analysis due to low turnout (6 attendees) and resultant protocol deviation. Of the 1122 event attendees, 428 participated at purposively sampled sites. Of those, 386 attendees (90%) consented to participate in research (minus the 6 removed from analysis; Figure), and 232 of 367 attendees (63%) who provided accurate contact information completed follow-up telephone interviews. The mean (SD) follow-up for the 220 participant interviews (Figure) was 5.4 (1.8) months (median, 4.8 months; interquartile range, 4.0-6.5 months).

**Figure.**
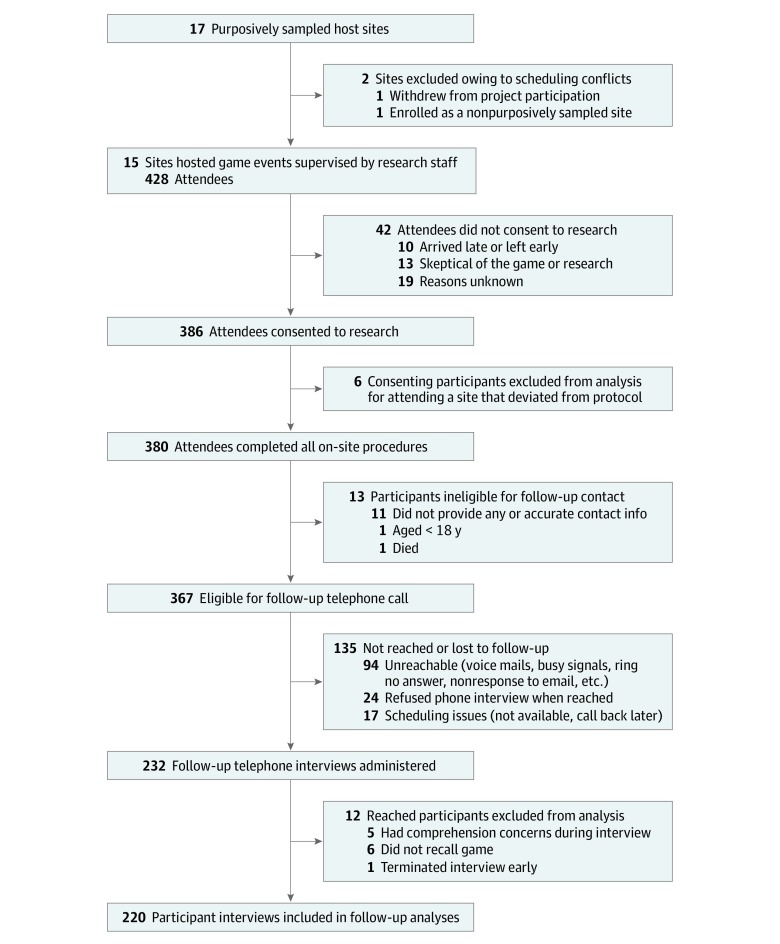
Participant Flow Diagram of Purposively Sampled Sites

### Characteristics of Participants

Participants’ mean (SD) age was 62.2 (13.8) years, with 304 of 380 participants (80%) being female and 348 of 380 (92%) being African American (Table 2). The characteristics of the participants are also shown by site (eTable 2 in the Supplement), demographic characteristics (urban vs rural), and region (eTable 3 in the Supplement).

**Table 2.** Characteristics of 380 Participants From Purposively Sampled Sites^a^^,^^b^

Characteristic	No. (%) of participants		
	Total sample (n = 380)	Completed follow-up (n = 220)	Did not complete follow-up (n = 160)
Sex			
Male	74 (19)	31 (14)	43 (27)
Female	304 (80)	189 (86)	115 (72)
No answer	2 (1)	0	2 (1)
Age, mean (SD), y	62.2 (13.8)^c^	62.6 (12.7)^d^	61.6 (15.2)^c^
Race/ethnicity			
African American	348 (92)	205 (93)	143 (89)
Native American including Native Indian	1 (0)	1 (0)	0
Hispanic or Latino	1 (0)	0	1 (0)
White	12 (3)	6 (3)	6 (4)
Asian or Pacific Islander	2 (1)	2 (1)	0
Other, multiple races indicated	11 (3)	5 (2)	6 (4)
No answer	5 (1)	1 (0)	4 (3)
Annual income, $			
≤10 000	67 (18)	36 (16)	31 (19)
20 000	57 (15)	32 (15)	25 (16)
30 000	57 (15)	34 (15)	23 (14)
40 000	35 (9)	22 (10)	13 (8)
50 000	25 (7)	16 (7)	9 (6)
>50 000	66 (17)	41 (19)	25 (16)
No answer	73 (19)	39 (18)	34 (21)
Marital status			
Married, engaged, or common-law	122 (32)	58 (26)	64 (40)
Single	130 (34)	72 (33)	58 (36)
Divorced or separated	67 (18)	52 (24)	15 (9)
Widowed	57 (15)	36 (16)	21 (13)
No answer	4 (1)	2 (1)	2 (1)
Highest level of educational attainment			
Did not finish high school	32 (8)	13 (6)	19 (12)
High school	77 (20)	38 (17)	39 (24)
Some college	115 (30)	68 (31)	47 (29)
Associate’s degree	29 (8)	20 (9)	9 (6)
Bachelor’s degree	55 (14)	34 (15)	21 (13)
Graduate degree	70 (18)	46 (21)	24 (15)
No answer	2 (1)	1 (0)	1 (1)
How important is religion or spirituality in your life?			
Extremely important	265 (70)	158 (72)	107 (67)
Very important	90 (24)	51 (23)	39 (24)
Somewhat important	19 (5)	7 (3)	12 (8)
Not very important	2 (1)	1 (0)	1 (1)
Not important at all	1 (0)	1 (0)	0
No answer	3 (1)	2 (1)	1 (1)
Religious affiliation			
Protestant Baptist	165 (43)	96 (44)	69 (43)
Protestant, other or not specified	114 (30)	71 (32)	43 (27)
Catholic	47 (12)	26 (12)	21 (13)
Muslim	3 (1)	2 (1)	1 (1)
Jewish	0	0	0
Jehovah’s Witness	3 (1)	2 (1)	1 (1)
Hindu	1 (0)	1 (0)	0
Agnostic	2 (1)	2 (1)	0
Atheist	0	0	0
Other	13 (3)	3 (1)	10 (6)
None	4 (1)	1 (0)	3 (2)
Prefer not to answer	13 (3)	8 (4)	5 (3)
No answer	15 (4)	8 (4)	7 (4)
Made major medical decisions (ie, life or death) for another person in the past 5 y?			
Yes	106 (28)	64 (29)	42 (26)
No	266 (70)	153 (70)	113 (71)
No answer	8 (2)	3 (1)	5 (3)
How would you say your health is in general?			
Excellent	33 (9)	16 (7)	17 (11)
Very good	188 (49)	112 (51)	76 (48)
Fair	147 (39)	87 (40)	60 (38)
Poor	7 (2)	3 (1)	4 (3)
Very poor	2 (1)	1 (0)	1 (1)
No answer	3 (1)	1 (0)	2 (1)
No. of hospital admissions in the past 5 y			
0	202 (53)	123 (56)	79 (49)
1-2	129 (34)	73 (33)	56 (35)
3-5	30 (8)	14 (6)	16 (10)
≥6	9 (2)	4 (2)	5 (3)
No answer	10 (3)	6 (3)	4 (3)
Need assistance with tasks (select all that apply)			
Washing, bathing, eating, taking medications, ambulation	15 (4)	5 (2)	10 (6)
Shopping or going on social outings	26 (7)	13 (6)	13 (8)
Going to health care visits	22 (6)	8 (4)	14 (9)
Organizing finances	35 (9)	22 (10)	13 (8)
Other	12 (3)	7 (3)	5 (3)
None or no response	311 (82)	184 (84)	127 (79)
Diagnoses (select all that apply)			
Dementia	3 (1)	0	3 (2)
Cancer	37 (10)	21 (10)	16 (10)
Heart or vascular disease	94 (25)	62 (28)	32 (20)
Lung disease (other than cancer)	14 (4)	9 (4)	5 (3)
Diabetes	115 (30)	71 (32)	44 (28)
Kidney disease	13 (3)	8 (4)	5 (3)
Other	35 (9)	26 (12)	9 (6)
None or no response	155 (41)	81 (37)	74 (46)
Autoimmune disease	13 (3)	7 (3)	6 (4)

^a^ See eAppendix 3 in the Supplement for additional details on data coding and classification.

^b^ Excluded 6 attendees from 1 site due to low attendance resulting in protocol deviation.

^c^ Missing 9 attendees.

^d^ Missing 10 attendees.

### Acceptability Outcomes

The calculated NPS was positive for all sites, ranging from 5.88 to 90.91, with an overall score of 57.89 (Table 3). The overall mean (SD) raw NPS score was 8.76 (2.02), with the mean score by sites ranging from 7.21 to 9.75. The mean (SD) conversation satisfaction score was 6.21 (0.93), and mean (SD) site scores ranged from 5.58 (0.98) to 6.88 (0.32). The mean (SD) conversation realism score was 5.20 (1.01), and the mean (SD) site scores ranged from 4.86 (1.27) to 5.40 (1.54) (eTable 4 in the Supplement).

**Table 3.** Rates of ACP Behavior Among 220 Participants

**ACP behavior**^**a**^	**Participants, No./total No. (%)**
Completed new advance directive	91/220 (41)
Updated, reread, or completed new advance directive	106/220 (48)
Talked to loved ones	176/220 (80)
Talked to clinician	43/220 (20)
Discussed game	154/220 (70)
Reviewed resources	122/219 (56)
Funeral planning^b^	14/212 (6)
Financial or insurance planning^b^	20/212 (9)
Other behavior	14/212 (6)
≥1 ACP behavior	214/219 (98)
≥3 ACP behaviors	145/215 (67)

Abbreviation: ACP, advance care planning.

^a^ In calculating the number of ACP behaviors performed by the time of the follow-up telephone call, missing data depended on the behavior being assessed and the responses that were given. For example, when calculating the rate of respondents who performed at least 1 ACP behavior, if participants indicated at least 1 behavior, their responses to the remaining behaviors were not considered. However, if there was no indication of a behavior, and the participant did not provide a definitive yes or no response to some behaviors, then it was considered unknown as to whether at least 1 behavior was completed; thus, it was treated as missing a response for “at least 1 completed behavior.” Similar coding logic was used to assess the completion of at least 3 ACP behaviors.

^b^ Categories emerged from open-ended behavior prompt.

### AD Completion Rates and ACP Behaviors

Table 3 gives rates of ACP behaviors reported at the follow-up telephone call. Of 220 participants, 68 (31%) reported having had an AD prior to the game, 91 (41%) completed a new AD, and 106 (48%) completed a new AD or revised an existing AD. Furthermore, 176 (80%) discussed end-of-life issues with loved ones, 214 of 219 (98%) completed at least 1 ACP behavior, and 145 of 215 (67%) completed 3 or more ACP behaviors. Scores on all domains of the ACP Engagement Survey increased (Table 4 and eTable 5 in the Supplement). There was a moderate and significant increase in the self-efficacy domain (mean [SD] difference before and after the game, 0.54 [0.98]; *P* < .001) and a small but significant increase in the knowledge (difference, 0.38 [1.24]; *P* < .001) and readiness (difference, 0.33 [0.98]; *P* < .001) domains as well as in the total score (difference, 0.40 [0.74]; *P* < .001). Behavioral rates and scores on the ACP Engagement Survey had no consistent patterns by site, demographic characteristics, or region (eTable 6 and eTable 7 in the Supplement).

**Table 4.** ACP Engagement Survey (34-Item) Results From 220 Participants^**a**^

Domain	Paired No.	Mean (SD) score		Score difference (after − before)	*P* value
		Before game	After game		
Knowledge (2 items)	210	3.63 (1.15)	4.01 (0.96)	0.38 (1.24)	<.001
Contemplation (3 items)	196	3.24 (1.13)	3.36 (0.98)	0.12 (1.12)	.16
Self-efficacy (12 items)	173	3.64 (1.01)	4.18 (0.81)	0.54 (0.98)	<.001
Readiness (17 items)	164	2.86 (1.09)	3.19 (0.90)	0.33 (0.98)	<.001
Total score (34 items)	144	3.19 (0.92)	3.59 (0.73)	0.40 (0.74)	<.001
ACP Engagement Survey 4-item^b^	195	2.95 (1.17)	3.29 (0.98)	0.33 (1.03)	<.001

Abbreviation: ACP, advance care planning.

^a^ Moderate effect size ranges from 0.50 to 0.79.

^b^ The 4-item version of the ACP-Engagement Survey includes only readiness domain items and was extracted from the full 34-item version.

### Themes From Telephone Interviews

Five major themes emerged from participants playing the game (eTable 5 in the Supplement): (1) it was a safe, fun, and enjoyable context for engaging in ACP conversations; (2) it offered new information and perspectives; (3) it was emotionally beneficial; (4) it increased appreciation for both the value and the need for ACP; and (5) it empowered and motivated performance of ACP behaviors. Additional subthemes and representative quotes are given in eTable 5 and eTable 8 in the Supplement.

A joint display that aligns the quantitative and qualitative results in accordance with this convergent, mixed-methods study is given in eTable 5 in the Supplement.^43^ We found that the data consistently converged in all 3 constructs of interest: satisfaction with the game, acceptability and endorsement of the experience, and self-efficacy and motivation for behavioral change.

## Discussion

This national study showed that a low-cost ($2.50/participant) and scalable game intervention may offer a feasible and acceptable approach for engaging underserved African American populations in ACP. Finding new and innovative ways to engage this hard-to-reach community in ACP is a critical first step toward reducing health disparities associated with end-of-life care for underserved populations.^1^ To our knowledge, this is the largest community-based dissemination of an ACP intervention among underserved African American communities. Our data indicated that the game events were well attended and highly endorsed. These data suggest that the game intervention was not only feasible to implement but also acceptable in African American communities, in which reticence for discussing end-of-life issues has been well documented.^7,18,22^

Unlike traditional approaches involving in-person interactions with health care professionals,^20^ we used a pragmatic delivery model that leveraged community networks. Such an approach is particularly appropriate in communities that may distrust or are less likely to use the health care system. Our qualitative data suggest that participants appreciated having the activity hosted within their social and faith-based communities, which in turn provided opportunities to share and learn from the experiences of trusted peers. Both of these findings highlight the value of addressing ACP within community networks via a trusted community venue (eg, hosting events in places of worship and community centers). This may be particularly salient within African American communities with strong reliance on social networks for information dissemination. The high levels of community engagement may be explained by our community-based delivery model because it sidestepped the need to interact with a distrusted health care system, an approach that has been successfully modeled in other health care initiatives using barbershops.^44^ Thus, our model using trusted community organizations might be used for health care initiatives beyond ACP whose goal is to engage underserved communities in important health behaviors.

Our qualitative data also suggested that the game itself may be associated with the success observed in this project. Numerous studies have reported that discussions about death and dying are perceived as unpleasant, uncomfortable, or intimidating.^21,45,46^ The game overcomes this barrier by reframing these discussions as an enjoyable activity in which players share stories, laugh, and learn from one anothers’ experiences. Using a social, conversation game helps establish psychological safety—the shared belief that individuals in a group can bring up risky topics or ideas.^47^ Players consistently report that the game creates a safe, nonthreatening environment that supports sensitive conversations.^27,28,29^ Furthermore, because the game is engaging and enjoyable and promotes positive reinforcement from the group, it serves to motivate players that may facilitate follow-through with subsequent ACP behaviors.

It is notable that 80% of participants had end-of-life conversations with loved ones because, even in populations where ACP is more prevalent, rates of end-of-life discussions are only 40% to 60%.^16,19,48,49^ Although our study was not designed to assess the effectiveness of the intervention with regard to behavior, our finding that 41% of participants completed a new AD is encouraging given the less than 25% baseline rate of AD completion among African American individuals^1,16,17,18,19,48,50,51,52^ and the much lower rates (13%) reported in other studies with underserved populations.^49^ That said, the secondary outcome of change in score on the ACP intervention was low in some domains, and the effect sizes on this instrument were small to moderate. Furthermore, in mixed-methods data reported separately, participants reported a low level skepticism and positive attitudes about ACP in general (unpublished data, 2020).

Although the study was not designed to compare findings across sites, similar rates of behavioral performance and levels of satisfaction and endorsement were observed regardless of site, demographic characteristics, and region. This suggests that use of a serious game may translate well across varied geographic settings.

### Limitations and Strengths

Given the national scope and the community-based nature of data collection, outcomes relied on self-report, leaving open the potential for social desirability bias and overreporting of ACP behaviors. Visual verification of AD completion would be useful in future studies. Furthermore, in the absence of a randomized clinical trial, it was not possible to infer or speak directly to any causation between the game and the ACP behaviors that followed. To manage potential researcher bias, research assistants with no relationship with the game’s producer collected the data. Finally, our study included predominantly female participants and African American participants, thus findings may not be generalizable to other populations.

Despite these limitations, this study has several strengths. First, this was a national sample with high rate of recruitment in a population that is traditionally hard to reach. Second, to our knowledge, this project is among the largest to evaluate an ACP intervention in so many communities and regions of the US. Third, the study protocol and analyses closely followed the National Institutes of Health best practices for mixed-methods health research, and our qualitative procedures adhered to published guidelines of methodologic rigor.^53,54,55,56^ Fourth, the consistent and highly convergent quantitative and qualitative data integration increased the validity and reliability of findings.^57,58,59^

## Conclusions

This project successfully engaged a nationwide audience of underserved communities in ACP. The present findings suggest that a serious game may be a feasible and well-received intervention in African American communities. As a low-cost and pragmatic intervention for increasing ACP engagement in underserved African American communities, such a game may help reduce health disparities associated with end-of-life care. Randomized clinical trials are needed to assess its effect on ACP behavioral performance and actual end-of-life care.

## Notes

Supplement. **eAppendix 1.** Additional Procedures and Data Collection **eAppendix 2.** Open-ended Phone Interview Questions **eAppendix 3.** Analytic Procedural Notes **eTable 1.** Event Agenda **eTable 2.** Participant Characteristics by Site **eTable 3.** Participant Characteristics by Demographic and Region **eTable 4.** ACP Behavior Rates by Site, Demographic, and Region **eTable 5.** Mixed-Methods Joint Display **eTable 6.** ACP Engagement Survey Results by Site, Demographic, and Region **eTable 7.** Net Promoter Score, Conversation Satisfaction, and Conversation Realism Results by Site, Demographic, and Region **eTable 8.** Additional Qualitative Quotations from Phone Interviews
