# Supplementary material for: Association of Participation in an End-of-Life Conversation Game With Advance Care Planning Behavior and Perspectives Among African American Individuals
Source: JAMA Netw Open. 2020 May 8;3(5):e204315. doi: 10.1001/jamanetworkopen.2020.4315 (PMC7210487; doi:10.1001/jamanetworkopen.2020.4315)
Supplement: Supplement. — eAppendix 1. Additional Procedures and Data Collection eAppendix 2. Open-ended Phone Interview Questions eAppendix 3. Analytic Procedural Notes eTable 1. Event Agenda eTable 2. Participant Characteristics by Site eTable 3. Participant Characteristics by Demographic and Region eTable 4. ACP Behavior Rates by Site, Demographic, and Region eTable 5. Mixed-Methods Joint Display eTable 6. ACP Engagement Survey Results by Site, Demographic, and Region eTable 7. Net Promoter Score, Conversation Satisfaction, and Conversation Realism Results by Site, Demographic, and Region eTable 8. Additional Qualitative Quotations from Phone Interviews [file jamanetwopen-3-e204315-s001.pdf]

Supplementary Online Content

Van Scoy LJ, Levi BH, Witt P, et al. Association of participation in an end-of-life conversation game with advance care planning behavior and perspectives among African American individuals. *JAMA Netw Open*. 2020;3(5):e204315. doi:10.1001/jamanetworkopen.2020.4315

eAppendix 1. Additional Procedures and Data Collection
eAppendix 2. Open-ended Phone Interview Questions
eAppendix 3. Analytic Procedural Notes
eTable 1. Event Agenda
eTable 2. Participant Characteristics by Site
eTable 3. Participant Characteristics by Demographic and Region
eTable 4. ACP Behavior Rates by Site, Demographic, and Region
eTable 5. Mixed-Methods Joint Display
eTable 6. ACP Engagement Survey Results by Site, Demographic, and Region
eTable 7. Net Promoter Score, Conversation Satisfaction, and Conversation Realism Results by Site, Demographic, and Region
eTable 8. Additional Qualitative Quotations from Phone Interviews

This supplementary material has been provided by the authors to give readers additional information about their work.

© 2020 Van Scoy LJ et al. *JAMA Network Open*.

eAppendix 1. Additional Procedures and Data Collection

Procedures at Non-Purposively Sampled Sites
Events at non-purposively sampled sites followed the same script as the purposively sampled sites absent the on-site data collection procedures (questionnaires). Events were run by the host as there was no on-site research coordinator on the day of the event. Participants at non-sampled sites completed a basic demographic questionnaire as part of an optional, anonymous survey at the end of the event. Hosts also distributed opt-in cards that allowed participants at non-purposively sampled sites to opt-in to a brief interview via follow-up phone call. Opt-ins were self-addressed stamped postcards and returned to the research team.

Game Modifications
While not pre-specified during study design, this study explored a slightly modified version of the *Hello* game which altered the question order from the commercially available iteration of the game. This alternate version was explored to learn whether the game questions altered the game experience or the subsequent behavior rates. In determining blinded game version shipments (commercially available vs. modified) to various sites, a stratified allocation template (25 blocks of size 2) was used, according to site type (Purposively Sampled vs. Non-Purposively Sampled), demographics (rural vs. urban), and region (West, Northeastern, Midwest, South) – resulting in 16 different stratification combinations. Neither quantitative or qualitative analyses uncovered any meaningful differences although the study was not designed to make any definitive conclusions about either game version.

Qualitative Data Collection (Focus Groups)
A convenience sample of participants from game events at purposively sampled sites participated in focus groups that explored the perceptions of and opinions about the *Hello* game experience. The focus groups performed a needs assessment related to opinions about ACP in general, along with community needs for ACP resources. Data from these focus groups are reported separately.

© 2020 Van Scoy LJ et al. *JAMA Network Open*.

eAppendix 2. Open-ended Phone Interview Questions

All phone interviews were audio-recorded. After completing quantitative questionnaires (ACP Behavior questionnaire and ACP Engagement Survey), responses to the three open-ended questions were transcribed verbatim: 1) Talking about end-of-life issues can be very hard for many people. Why do you think this is?; 2) Tell me about your experience playing *Hello*; and 3) Please tell me how you think playing *Hello* has affected you.

© 2020 Van Scoy LJ et al. *JAMA Network Open*.

eAppendix 3. Analytic Procedural Notes

Classifications and Coding of Participant Characteristics for Purposively Sampled Sites
One participant under the age of 18 was enrolled and included in analysis. . For annual income, text responses were rounded and classified according to the reported categories (e.g. responses between $10,001 and $14,999 were categorized as $10,000 – responses between $15,000 and $19,999 were categorized as $20,000, etc.). Religious Affiliation was grouped into categories post-hoc. Protestant Baptist includes any listed indications of 'Baptist', including Christian-Baptist, Treewill Baptist, Baptist Congregational, Baptist AME, Baptist Non-Denominational, and/or Southern Baptist. Protestant Other/Not Specified includes indications of Apostolic, Holiness, Christian, Christian Non-Denominational, Christian Pentecostal, Church of Christ/God, Non-Denominational, Pentecostal, Methodist, Unity, Child of God, AME, Episcopalian, United Church of Christ, and/or Presbyterian. Tasks requiring assistance were collected via 'Select all that Apply' options, in addition to open-ended responses to an 'Other' option that may not have been listed. Final assistance classifications were decided post-hoc. Medical conditions were collected via 'Select all that Apply' options, in addition to open-ended responses to an 'Other' option that may not have been listed. Final condition classifications were decided post-hoc and can be described as follows: Heart and/or Vascular Disease includes indications of Heart Disease, A-Fib, Aphasia, High Blood Pressure/Hypertension, Hyperlipidemia, Congestive Heart Failure, High Cholesterol or Hypercholesterolemia, Stroke, and/or TIA. Lung Disease (other than cancer) includes indications of Lung Disease, Asthma, Bronchitis, and/or COPD. Diabetes includes indications of Pre-Diabetes. Autoimmune Disease includes indications of Autoimmune Disease, Hypothyroidism, Lupus, Rheumatoid Arthritis, Sarcoidosis, and/or Thyroid Disease. The 'Other' category consisted of many other conditions that the research team did not feel could be confidently coded as one of the previous classifications delineated.

Scale Error on the Net Promoter Score
Due to administrative error, the Net Promoter Score was captured on a 1-10 point Likert scale. While the 10 point scale has been used widely, the original validation studies utilize a 0-10 point Likert scale.

Varied Duration of Follow-up
We report the mean duration of follow-up as 5.4 months (median 4.8) and note that three participant interviews were conducted three days prior to the 90 day mark and ten participants had interviews completed beyond the 9 month timepoint due to scheduling issues. The longest reported follow-up was conducted 347 days after the event (11.4 months). Documented call dates where calls were made but non-reachable were not considered in the calculation of the follow-up response time. Days were converted to months dividing by 30.4375 (365.25/12).

© 2020 Van Scoy LJ et al. *JAMA Network Open*.

eTable 1. Event Agenda

Hello Game Day Event Agenda for Research Sites	Responsible Party
Welcome and introductions (5 min.)	Host
Informed consent for research participants (10 min.)	Research assistant
Complete pre-game questionnaires (30 min.)<br>Baseline characteristics<br>ACP Engagement Survey- 34 item version	Research assistant
Explain game rules (10 min.)	Host
Hello game-play (60 min.)	Host
Optional stretch break/snacks (10 min.)	Host
Explain focus groups (10 min.)	Research assistant
Complete post-game questionnaires (15 min.)<br>Conversation satisfaction<br>Conversation realism<br>Net Promoter Score	Research assistant
Conduct focus group(s) (45 min)<br>Lunch/refreshments for those opting out of focus group(s)	Research assistant
Distribute resource materials/handouts and gift cards (15 min.)	Research assistant
Dismissal (5 min.)	Host

© 2020 Van Scoy LJ et al. *JAMA Network Open*.

eTable 2. Participant Characteristics by Site

	AL[a]	CA[b]	DC[c]	FL[d]	GA[e]	IL[f]	LA[g]	MO[h]	NC[i]	NV[j]	NY[k]	PA[l]	TX[m]	WI[n]	Overall
	n=50	n=18	n=45	n=23	n=35	n=13	n=43	n=17	n=24	n=36	n=22	n=16	n=20	n=18	
**Age, mean (SD)**	59 (13)	65 (14)	66 (16)	63 (15)	67 (12)	74 (7)	62 (11)	59 (14)	56 (16)	68 (9)	52 (18)	54 (12)	62 (11)	64 (12)	62 (14)
No answer, No. (%)[o]	1 (0)	2 (1)	2 (0)	0 (0)	2 (1)	1 (0)	0 (0)	1 (1)	1 (0)	3 (1)	1 (0)	1 (1)	4 (2)	0 (0)	19 (1)
**Gender, No. (%)**															
Male	9 (18)	7 (39)	6 (13)	4 (17)	8 (23)	2 (15)	6 (14)	3 (18)	4 (17)	6 (17)	11 (50)	4 (25)	2 (10)	2 (11)	74 (19)
Female	40 (80)	11 (61)	39 (87)	19 (83)	26 (74)	11 (85)	37 (86)	14 (82)	20 (83)	30 (83)	11 (50)	12 (75)	18 (90)	16 (89)	304 (80)
No answer	1 (2)	0 (0)	0 (0)	0 (0)	1 (3)	0 (0)	0 (0)	0 (0)	0 (0)	0 (0)	0 (0)	0 (0)	0 (0)	0 (0)	2 (1)
**Race/ethnicity, No. (%)**															
African American	49 (98)	13 (72)	42 (93)	22 (96)	30 (86)	12 (92)	39 (91)	13 (76)	22 (92)	34 (94)	19 (86)	16 (100)	19 (95)	18 (100)	348 (92)
Native or American Indian	0 (0)	0 (0)	0 (0)	1 (4)	0 (0)	0 (0)	0 (0)	0 (0)	0 (0)	0 (0)	0 (0)	0 (0)	0 (0)	0 (0)	1 (0)
Hispanic or Latino	0 (0)	1 (6)	0 (0)	0 (0)	0 (0)	0 (0)	0 (0)	0 (0)	0 (0)	0 (0)	0 (0)	0 (0)	0 (0)	0 (0)	1 (0)
White	1 (2)	2 (11)	1 (2)	0 (0)	1 (3)	0 (0)	3 (7)	1 (6)	2 (8)	1 (3)	0 (0)	0 (0)	0 (0)	0 (0)	12 (3)
Asian or Pacific Islander	0 (0)	1 (6)	1 (2)	0 (0)	0 (0)	0 (0)	0 (0)	0 (0)	0 (0)	0 (0)	0 (0)	0 (0)	0 (0)	0 (0)	2 (1)
Other	0 (0)	1 (6)	1 (2)	0 (0)	3 (9)	1 (8)	1 (2)	3 (18)	0 (0)	0 (0)	1 (5)	0 (0)	0 (0)	0 (0)	11 (3)
No answer	0 (0)	0 (0)	0 (0)	0 (0)	1 (3)	0 (0)	0 (0)	0 (0)	0 (0)	1 (3)	2 (9)	0 (0)	1 (5)	0 (0)	5 (1)
**Annual Income, No. (%)**															
<$10,000	20 (40)	2 (11)	3 (7)	2 (9)	6 (17)	1 (8)	6 (14)	7 (41)	4 (17)	6 (17)	6 (27)	2 (13)	1 (5)	1 (6)	67 (17)
$20,000	7 (14)	2 (11)	8 (18)	4 (17)	8 (23)	3 (23)	6 (14)	0 (0)	4 (17)	8 (22)	0 (0)	4 (25)	2 (10)	1 (6)	57 (15)
$30,000	4 (8)	1 (6)	6 (13)	4 (17)	7 (20)	3 (23)	8 (19)	2 (12)	4 (17)	3 (8)	5 (23)	2 (13)	4 (20)	4 (22)	57 (15)
$40,000	3 (6)	2 (11)	4 (9)	1 (4)	2 (6)	0 (0)	4 (9)	0 (0)	4 (17)	4 (11)	2 (9)	1 (6)	3 (15)	5 (28)	35 (9)
$50,000	2 (4)	1 (6)	3 (7)	4 (17)	1 (3)	4 (31)	6 (14)	1 (6)	0 (0)	3 (8)	0 (0)	0 (0)	0 (0)	0 (0)	25 (7)
>$50,000	1 (2)	9 (50)	15 (33)	5 (22)	1 (3)	1 (8)	9 (21)	3 (18)	3 (13)	4 (11)	3 (14)	4 (25)	6 (30)	2 (11)	66 (17)

© 2020 Van Scoy LJ et al. *JAMA Network Open*.

	AL[a]	CA[b]	DC[c]	FL[d]	GA[e]	IL[f]	LA[g]	MO[h]	NC[i]	NV[j]	NY[k]	PA[l]	TX[m]	WI[n]	Overall
	n=50	n=18	n=45	n=23	n=35	n=13	n=43	n=17	n=24	n=36	n=22	n=16	n=20	n=18	
**Annual Income, No. (%) (continued)**															
No answer	13 (26)	1 (6)	6 (13)	3 (13)	10 (29)	1 (8)	4 (9)	4 (24)	5 (21)	8 (22)	6 (27)	3 (19)	4 (20)	5 (28)	73 (19)
**Marital status, No. (%)**															
Married	18 (36)	6 (33)	10 (22)	11 (48)	5 (14)	3 (23)	20 (47)	7 (41)	5 (21)	7 (19)	10 (45)	8 (50)	6 (30)	6 (33)	122 (32)
Single	21 (42)	4 (22)	17 (38)	7 (30)	14 (40)	4 (31)	12 (28)	6 (35)	13 (54)	7 (19)	8 (36)	6 (38)	8 (40)	3 (17)	130 (36)
Divorced	7 (14)	5 (28)	8 (18)	2 (9)	10 (29)	5 (38)	5 (12)	1 (6)	3 (13)	11 (31)	0 (0)	1 (6)	4 (20)	5 (28)	67 (18)
Widowed	3 (6)	3 (17)	10 (22)	3 (13)	4 (11)	1 (8)	6 (14)	3 (18)	3 (13)	10 (28)	4 (18)	1 (6)	2 (10)	4 (22)	57 (16)
No answer	1 (2)	0 (0)	0 (0)	0 (0)	2 (6)	0 (0)	0 (0)	0 (0)	0 (0)	1 (3)	0 (0)	0 (0)	0 (0)	0 (0)	4 (1)
**Highest level of education attainment, No. (%)**															
Did not finish high school	6 (12)	1 (6)	0 (0)	1 (4)	2 (6)	1 (8)	4 (9)	6 (35)	0 (0)	2 (6)	6 (27)	3 (19)	0 (0)	0 (0)	32 (8)
High school	24 (48)	2 (11)	6 (13)	0 (0)	5 (14)	1 (8)	3 (7)	0 (0)	8 (33)	11 (31)	7 (32)	6 (38)	0 (0)	4 (22)	77 (20)
Some college	13 (26)	6 (33)	12 (27)	7 (30)	17 (49)	4 (31)	16 (37)	3 (18)	8 (33)	14 (39)	4 (18)	2 (13)	5 (25)	4 (22)	115 (32)
Associate’s degree	1 (2)	2 (11)	4 (9)	3 (13)	1 (3)	0 (0)	2 (5)	1 (6)	0 (0)	5 (14)	3 (14)	2 (13)	1 (5)	4 (22)	29 (8)
Bachelor’s degree	4 (8)	5 (28)	9 (20)	3 (13)	4 (11)	3 (23)	9 (21)	3 (18)	4 (17)	2 (6)	0 (0)	0 (0)	6 (30)	3 (17)	55 (14)
Graduate degree	2 (4)	2 (11)	14 (31)	9 (39)	4 (11)	4 (31)	9 (21)	4 (24)	4 (17)	2 (6)	2 (9)	3 (19)	8 (40)	3 (17)	70 (18)
No answer	0 (0)	0 (0)	0 (0)	0 (0)	2 (6)	0 (0)	0 (0)	0 (0)	0 (0)	0 (0)	0 (0)	0 (0)	0 (0)	0 (0)	2 (1)
**How important is religion in your life? No. (%)**															
Extremely important	36 (72)	9 (50)	26 (58)	19 (83)	18 (51)	10 (77)	40 (93)	12 (71)	15 (63)	26 (72)	9 (41)	14 (88)	14 (70)	17 (94)	265 (70)
Very important	13 (26)	7 (39)	16 (36)	4 (17)	10 (29)	3 (23)	2 (5)	3 (18)	4 (17)	9 (25)	10 (45)	2 (13)	6 (30)	1 (6)	90 (24)
Somewhat important	1 (2)	2 (11)	2 (4)	0 (0)	4 (11)	0 (0)	1 (2)	1 (6)	5 (21)	1 (3)	2 (9)	0 (0)	0 (0)	0 (0)	19 (5)
Not very important	0 (0)	0 (0)	1 (2)	0 (0)	1 (3)	0 (0)	0 (0)	0 (0)	0 (0)	0 (0)	0 (0)	0 (0)	0 (0)	0 (0)	2 (1)

© 2020 Van Scoy LJ et al. *JAMA Network Open*.

	AL[a]	CA[b]	DC[c]	FL[d]	GA[e]	IL[f]	LA[g]	MO[h]	NC[i]	NV[j]	NY[k]	PA[l]	TX[m]	WI[n]	Overall
	n=50	n=18	n=45	n=23	n=35	n=13	n=43	n=17	n=24	n=36	n=22	n=16	n=20	n=18	
**How important is religion in your life? No. (%) (continued)**															
Not at all important	0 (0)	0 (0)	0 (0)	0 (0)	0 (0)	0 (0)	0 (0)	1 (6)	0 (0)	0 (0)	0 (0)	0 (0)	0 (0)	0 (0)	1 (0)
No answer	0 (0)	0 (0)	0 (0)	0 (0)	2 (6)	0 (0)	0 (0)	0 (0)	0 (0)	0 (0)	1 (5)	0 (0)	0 (0)	0 (0)	3 (1)
**Religious affiliation? No. (%)**															
Catholic	0 (0)	0 (0)	12 (27)	1 (4)	2 (6)	1 (8)	31 (72)	0 (0)	0 (0)	0 (0)	0 (0)	0 (0)	0 (0)	0 (0)	47 (12)
Protestant (Baptist)	37 (74)	8 (44)	11 (24)	16 (70)	10 (29)	5 (38)	11 (26)	9 (53)	13 (54)	15 (42)	5 (23)	2 (13)	6 (30)	17 (94)	165 (43)
Protestant (Other)	6 (12)	5 (28)	16 (36)	5 (22)	10 (29)	7 (54)	0 (0)	4 (24)	8 (33)	17 (47)	10 (45)	13 (81)	12 (60)	1 (6)	114 (30)
Muslim	0 (0)	0 (0)	0 (0)	0 (0)	1 (3)	0 (0)	0 (0)	0 (0)	0 (0)	1 (3)	1 (5)	0 (0)	0 (0)	0 (0)	3 (1)
Jehovah’s Witness	0 (0)	0 (0)	0 (0)	0 (0)	1 (3)	0 (0)	0 (0)	0 (0)	0 (0)	1 (3)	1 (5)	0 (0)	0 (0)	0 (0)	3 (1)
Hindu	0 (0)	0 (0)	1 (2)	0 (0)	0 (0)	0 (0)	0 (0)	0 (0)	0 (0)	0 (0)	0 (0)	0 (0)	0 (0)	0 (0)	1 (0)
Agnostic	0 (0)	0 (0)	1 (2)	0 (0)	0 (0)	0 (0)	0 (0)	1 (6)	0 (0)	0 (0)	0 (0)	0 (0)	0 (0)	0 (0)	2 (1)
Other	1 (2)	0 (0)	2 (4)	1 (4)	1 (3)	0 (0)	1 (2)	3 (18)	0 (0)	0 (0)	2 (9)	0 (0)	2 (10)	0 (0)	13 (3)
None	0 (0)	1 (6)	0 (0)	0 (0)	2 (6)	0 (0)	0 (0)	0 (0)	0 (0)	0 (0)	0 (0)	1 (6)	0 (0)	0 (0)	4 (1)
Prefer not to answer	2 (4)	3 (17)	2 (4)	0 (0)	3 (9)	0 (0)	0 (0)	0 (0)	3 (13)	0 (0)	0 (0)	0 (0)	0 (0)	0 (0)	13 (3)
No answer	4 (8)	1 (6)	0 (0)	0 (0)	5 (14)	0 (0)	0 (0)	0 (0)	0 (0)	2 (6)	3 (14)	0 (0)	0 (0)	0 (0)	15 (4)
**Have you ever had to make a major medical decision (i.e., life/death) for another person in the past 5 years? No. (%)**															
Yes	10 (20)	2 (11)	14 (31)	3 (13)	11 (31)	5 (38)	10 (23)	5 (29)	7 (29)	10 (28)	8 (36)	6 (38)	8 (40)	7 (39)	106 (28)
No	39 (78)	15 (83)	31 (69)	20 (87)	23 (66)	8 (62)	32 (74)	12 (71)	16 (67)	25 (69)	12 (55)	10 (63)	12 (60)	11 (61)	266 (70)
No answer	1 (2)	1 (6)	0 (0)	0 (0)	1 (3)	0 (0)	1 (2)	0 (0)	1 (4)	1 (3)	2 (9)	0 (0)	0 (0)	0 (0)	8 (2)
**How would you say your health is in general? No. (%)**															
Excellent	4 (8)	3 (17)	4 (9)	0 (0)	4 (11)	1 (8)	5 (12)	3 (18)	3 (13)	1 (3)	4 (18)	1 (6)	0 (0)	0 (0)	33 (9)
Very good	23 (46)	4 (22)	25 (56)	18 (78)	14 (40)	10 (77)	24 (56)	9 (53)	6 (25)	18 (50)	6 (27)	8 (50)	13 (65)	10 (56)	188 (49)

© 2020 Van Scoy LJ et al. *JAMA Network Open*.

	AL[a]	CA[b]	DC[c]	FL[d]	GA[e]	IL[f]	LA[g]	MO[h]	NC[i]	NV[j]	NY[k]	PA[l]	TX[m]	WI[n]	Overall
	n=50	n=18	n=45	n=23	n=35	n=13	n=43	n=17	n=24	n=36	n=22	n=16	n=20	n=18	
**How would you say your health is in general? No. (%) (continued)**															
Fair	22 (44)	10 (56)	15 (33)	5 (22)	17 (49)	2 (15)	13 (30)	3 (18)	14 (58)	16 (44)	10 (45)	6 (38)	7 (35)	7 (39)	147 (39)
Poor	1 (2)	1 (6)	1 (2)	0 (0)	0 (0)	0 (0)	0 (0)	1 (6)	0 (0)	1 (3)	1 (5)	1 (6)	0 (0)	0 (0)	7 (2)
Very Poor	0 (0)	0 (0)	0 (0)	0 (0)	0 (0)	0 (0)	0 (0)	1 (6)	0 (0)	0 (0)	1 (5)	0 (0)	0 (0)	0 (0)	2 (1)
No answer	0 (0)	0 (0)	0 (0)	0 (0)	0 (0)	0 (0)	1 (2)	0 (0)	1 (4)	0 (0)	0 (0)	0 (0)	0 (0)	1 (6)	3 (1)
**How many times have you been admitted to the hospital in the past 5 years? No. (%)**															
0 times	26 (52)	11 (61)	22 (49)	11 (48)	19 (54)	6 (46)	33 (77)	6 (35)	15 (63)	21 (58)	7 (32)	7 (44)	10 (50)	8 (44)	202 (53)
1-2 times	19 (38)	3 (17)	16 (36)	7 (30)	10 (29)	6 (46)	7 (16)	8 (47)	7 (29)	11 (31)	10 (45)	5 (31)	10 (50)	10 (56)	129 (34)
3-5 times	3 (6)	3 (17)	5 (11)	5 (22)	2 (6)	1 (8)	1 (2)	2 (12)	1 (4)	3 (8)	2 (9)	2 (13)	0 (0)	0 (0)	30 (8)
≥6 times	2 (4)	0 (0)	2 (4)	0 (0)	1 (3)	0 (0)	0 (0)	1 (6)	0 (0)	1 (3)	2 (9)	0 (0)	0 (0)	0 (0)	9 (2)
No answer	0 (0)	1 (6)	0 (0)	0 (0)	3 (9)	0 (0)	2 (5)	0 (0)	1 (4)	0 (0)	1 (5)	2 (13)	0 (0)	0 (0)	10 (3)
**Needing help with any of the following tasks? No. (%)**															
Washing	2 (4)	2 (11)	0 (0)	0 (0)	2 (6)	0 (0)	1 (2)	3 (18)	2 (8)	1 (3)	1 (5)	0 (0)	1 (5)	0 (0)	15 (4)
Shopping	3 (6)	3 (17)	2 (4)	0 (0)	3 (9)	1 (8)	1 (2)	3 (18)	2 (8)	4 (11)	2 (9)	0 (0)	0 (0)	2 (11)	26 (7)
Visiting MD	4 (8)	3 (17)	1 (2)	0 (0)	3 (9)	1 (8)	1 (2)	2 (12)	1 (4)	4 (11)	1 (5)	0 (0)	0 (0)	1 (6)	22 (6)
Finances	1 (2)	4 (22)	4 (9)	1 (4)	3 (9)	1 (8)	3 (7)	1 (6)	5 (21)	3 (8)	2 (9)	5 (31)	0 (0)	2 (11)	35 (9)
Other	1 (2)	2 (11)	3 (7)	0 (0)	2 (6)	0 (0)	1 (2)	2 (12)	1 (4)	0 (0)	0 (0)	0 (0)	0 (0)	0 (0)	12 (3)
None	43 (86)	12 (67)	36 (80)	22 (96)	28 (80)	11 (85)	39 (91)	13 (76)	15 (63)	30 (83)	17 (77)	11 (69)	19 (95)	15 (83)	311 (82)
**Have you been diagnosed with any of the following medical conditions? No. (%)**															
Dementia	1 (2)	0 (0)	1 (2)	0 (0)	0 (0)	0 (0)	0 (0)	0 (0)	0 (0)	0 (0)	0 (0)	0 (0)	1 (5)	0 (0)	3 (1)
Cancer	2 (4)	0 (0)	4 (9)	5 (22)	2 (6)	2 (15)	6 (14)	2 (12)	3 (13)	5 (14)	2 (9)	2 (13)	1 (5)	1 (6)	37 (10)
Heart /Vascular	9 (18)	6 (33)	12 (27)	4 (17)	8 (23)	3 (23)	13 (30)	8 (47)	4 (17)	12 (33)	3 (14)	3 (19)	5 (25)	4 (22)	94 (25)

© 2020 Van Scoy LJ et al. *JAMA Network Open*.

	AL[a]	CA[b]	DC[c]	FL[d]	GA[e]	IL[f]	LA[g]	MO[h]	NC[i]	NV[j]	NY[k]	PA[l]	TX[m]	WI[n]	Overall
	n=50	n=18	n=45	n=23	n=35	n=13	n=43	n=17	n=24	n=36	n=22	n=16	n=20	n=18	
**Have you been diagnosed with any of the following medical conditions? No. (%) (continued)**															
Lung	1 (2)	0 (0)	1 (2)	0 (0)	2 (6)	1 (8)	1 (2)	1 (6)	0 (0)	1 (3)	1 (5)	2 (13)	1 (5)	2 (11)	14 (4)
Diabetes	22 (44)	3 (17)	11 (24)	12 (52)	7 (20)	2 (15)	8 (19)	7 (41)	5 (21)	14 (39)	5 (23)	5 (31)	8 (40)	6 (33)	115 (30)
Kidney	4 (8)	0 (0)	0 (0)	3 (13)	0 (0)	1 (8)	1 (2)	1 (6)	0 (0)	3 (8)	0 (0)	0 (0)	0 (0)	0 (0)	13 (4)
Other	5 (10)	3 (17)	6 (13)	2 (9)	1 (3)	0 (0)	4 (9)	1 (6)	4 (17)	2 (6)	2 (9)	1 (6)	0 (0)	4 (22)	35 (9)
None	21 (42)	8 (44)	18 (40)	4 (17)	19 (54)	4 (31)	21 (49)	6 (35)	13 (54)	9 (25)	12 (55)	3 (19)	10 (50)	7 (39)	155 (41)
Autoimmune	0 (0)	1 (6)	4 (9)	0 (0)	1 (3)	0 (0)	3 (7)	0 (0)	0 (0)	1 (3)	0 (0)	2 (13)	0 (0)	1 (6)	13 (3)

[a] Livingston, Alabama, [b] Palo Alto, California, [c] Washington, DC, [d] Lakeland, Florida, [e] Atlanta, Georgia, [f] Chicago, Illinois, [g] Broussard, Louisiana, [h] St. Louis, Missouri, [i] Asheville, North Carolina, [j] Las Vegas, Nevada, [k] Sodus, New York, [l] Philadelphia, Pennsylvania, [m] Amarillo, Texas, [n] Milwaukee, Wisconsin
o Reported percentages represented as column percentages

© 2020 Van Scoy LJ et al. *JAMA Network Open*.

eTable 3. Participant Characteristics by Demographic and Region

	Urban	Rural	Northeast	Southern	Midwestern	Western	Total
	n=241	n=139	n=83	n=195	n=48	n=54	n=380
**Age, mean (SD)**	65 (13)	58 (14)	60 (17)	61 (13)	65 (13)	67 (11)	62 (14)
No answer, No. (%) [a]	16 (1)	3 (0)	4 (0)	8 (0)	2 (0)	5 (1)	19 (1)
**Gender, No. (%)**							
Male	44 (18)	30 (22)	21 (25)	33 (17)	7 (15)	13 (24)	74 (19)
Female	196 (81)	108 (78)	62 (75)	160 (82)	41 (85)	41 (76)	304 (80)
No answer	1 (0)	1 (0)	0 (0)	2 (1)	0 (0)	0 (0)	2 (1)
**Race/ethnicity, No. (%)**							
African American	219 (91)	129 (93)	77 (93)	181 (93)	43 (90)	47 (87)	348 (92)
Native-American Indian	1 (0)	0 (0)	0 (0)	1 (1)	0 (0)	0 (0)	1 (0)
Hispanic or Latino	1 (0)	0 (0)	0 (0)	0 (0)	0 (0)	1 (2)	1 (0)
White	6 (2)	6 (4)	1 (1)	7 (4)	1 (2)	3 (6)	12 (3)
Asian or Pacific Islander	2 (1)	0 (0)	1 (1)	0 (0)	0 (0)	1 (2)	2 (1)
Other	9 (4)	2 (1)	2 (2)	4 (2)	4 (8)	1 (2)	11 (3)
No answer	3 (1)	2 (1)	2 (2)	2 (1)	0 (0)	1 (2)	5 (1)
**Annual income, No. (%)**							
<$10,000	31 (13)	36 (26)	11 (13)	39 (20)	9 (19)	8 (15)	67 (18)
$20,000	40 (17)	17 (12)	12 (14)	31 (16)	4 (8)	10 (19)	57 (15)
$30,000	36 (15)	21 (15)	13 (16)	31 (16)	9 (19)	4 (7)	57 (15)
$40,000	22 (9)	13 (9)	7 (8)	17 (9)	5 (10)	6 (11)	35 (9)
$50,000	17 (7)	8 (6)	3 (4)	13 (7)	5 (10)	4 (7)	25 (7)
>$50,000	50 (21)	16 (12)	22 (27)	25 (13)	6 (13)	13 (24)	66 (17)
No answer	45 (19)	28 (20)	15 (18)	39 (20)	10 (21)	9 (17)	73 (19)
**Marital status, No. (%)**							
Married	69 (29)	53 (38)	28 (34)	65 (33)	16 (33)	13 (24)	122 (32)
Single	76 (32)	54 (39)	31 (37)	75 (38)	13 (27)	11 (20)	130 (34)
Divorced	52 (22)	15 (11)	9 (11)	31 (16)	11 (23)	16 (30)	67 (18)
Widowed	41 (17)	16 (12)	15 (18)	21 (11)	8 (17)	13 (24)	57 (15)
No answer	3 (1)	1 (1)	0 (0)	3 (2)	0 (0)	1 (2)	4 (1)
**Highest level of education attainment, No. (%)**							
Did not finish high school	16 (7)	16 (12)	9 (11)	13 (7)	7 (15)	3 (6)	32 (8)
High school	35 (15)	42 (30)	19 (23)	40 (21)	5 (10)	13 (24)	77 (20)
Some college	74 (31)	41 (30)	18 (22)	66 (34)	11 (23)	20 (37)	115 (30)
Associate’s degree	23 (10)	6 (4)	9 (11)	8 (4)	5 (10)	7 (13)	29 (8)
Bachelor’s degree	38 (16)	17 (12)	9 (11)	30 (15)	9 (19)	7 (13)	55 (14)
Graduate degree	53 (22)	17 (12)	19 (23)	36 (18)	11 (23)	4 (7)	70 (18)
No answer	2 (1)	0 (0)	0 (0)	2 (1)	0 (0)	0 (0)	2 (1)
**How important is religion in your life? No. (%)**							
Extremely important	165 (68)	100 (72)	49 (59)	142 (73)	39 (81)	35 (65)	265 (70)
Very important	61 (25)	29 (21)	28 (34)	39 (20)	7 (15)	16 (30)	90 (24)

© 2020 Van Scoy LJ et al. *JAMA Network Open*.

	Urban	Rural	Northeast	Southern	Midwestern	Western	Total
Somewhat important	10 (4)	9 (6)	4 (5)	11 (6)	1 (2)	3 (6)	19 (5)
	**Urban**	**Rural**	**Northeast**	**Southern**	**Midwestern**	**Western**	**Total**
	n=241	n=139	n=83	n=195	n=48	n=54	n=380
**How important is religion in your life? No. (%) (continued)**							
Not very important	2 (1)	0 (0)	1 (1)	1 (1)	0 (0)	0 (0)	2 (1)
Not at all important	1 (0)	0 (0)	0 (0)	0 (0)	1 (2)	0 (0)	1 (0)
No answer	2 (1)	1 (1)	1 (1)	2 (1)	0 (0)	0 (0)	3 (1)
**Religious Affiliation, No. (%)**							
Catholic	16 (7)	31 (22)	12 (14)	34 (17)	1 (2)	0 (0)	47 (12)
Protestant (Baptist)	99 (41)	66 (47)	18 (22)	93 (48)	31 (65)	23 (43)	165 (43)
Protestant (Other)	90 (37)	24 (17)	39 (47)	41 (21)	12 (25)	22 (41)	114 (30)
Muslim	2 (1)	1 (1)	1 (1)	1 (1)	0 (0)	1 (2)	3 (1)
Jehovah's Witness	2 (1)	1 (1)	1 (1)	1 (1)	0 (0)	1 (2)	3 (1)
Hindu	1 (0)	0 (0)	1 (1)	0 (0)	0 (0)	0 (0)	1 (0)
Agnostic	2(1)	0 (0)	1 (1)	0 (0)	1 (2)	0 (0)	2 (1)
Other	9 (4)	4 (3)	4 (5)	6 (3)	3 (6)	0 (0)	13 (3)
None	4 (2)	0 (0)	1 (1)	2 (1)	0 (0)	1 (2)	4 (1)
Prefer not to answer	8 (3)	5 (4)	2 (2)	8 (4)	0 (0)	3 (6)	13 (3)
No answer	8 (3)	7 (5)	3 (4)	9 (5)	0 (0)	3 (6)	15 (4)
**Have you had to make major medical decisions (i.e. life/death) for another person in the past 5 years? No. (%)**							
Yes	71 (29)	35 (25)	28 (34)	49 (25)	17 (35)	12 (22)	106 (28)
No	167 (69)	99 (71)	53 (64)	142 (73)	31 (65)	40 (74)	266 (70)
No answer	3 (1)	5 (4)	2 (2)	4 (2)	0 (0)	2 (4)	8 (2)
**How would you say your health is in general? No. (%)**							
Excellent	17 (7)	16 (12)	9 (11)	16 (8)	4 (8)	4 (7)	33 (9)
Very good	129 (54)	59 (42)	39 (47)	98 (50)	29 (60)	22 (41)	188 (49)
Fair	88 (37)	59 (42)	31 (37)	78 (40)	12 (25)	26 (48)	147 (39)
Poor	5 (2)	2 (1)	3 (4)	1 (1)	1 (2)	2 (4)	7 (2)
Very Poor	1 (0)	1 (1)	1 (1)	0 (0)	1 (2)	0 (0)	2 (1)
No answer	1 (0)	2 (1)	0 (0)	2 (1)	1 (2)	0 (0)	3 (1)
**How many times have you been admitted to the hospital in the past 5 years? No. (%)**							
0 times	121 (50)	81 (58)	36 (43)	114 (58)	20 (42)	32 (59)	202 (53)
1-2 times	86 (36)	43 (31)	31 (37)	60 (31)	24 (50)	14 (26)	129 (34)
3-5 times	23 (10)	7 (5)	9 (11)	12 (6)	3 (6)	6 (11)	30 (8)
≥6 times	5 (2)	4 (3)	4 (5)	3 (2)	1 (2)	1 (2)	9 (2)
No answer	6 (2)	4 (3)	3 (4)	6 (3)	0 (0)	1 (2)	10 (3)
**Needing help with the following tasks? No. (%)**							
Washing	9 (4)	6 (4)	1 (1)	8 (4)	3 (6)	3 (6)	15 (4)
Shopping	18 (7)	8 (6)	4 (5)	9 (5)	6 (13)	7 (13)	26 (7)
Visiting MD	15 (6)	7 (5)	2 (2)	9 (5)	4 (8)	7 (13)	22 (6)
Finances	24 (10)	11 (8)	11 (13)	13 (7)	4 (8)	7 (13)	35 (9)

© 2020 Van Scoy LJ et al. *JAMA Network Open*.

Other	9 (4)	3 (2)	3 (4)	5 (3)	2 (4)	2 (4)	12 (3)
None	197 (82)	114 (82)	64 (77)	166 (85)	39 (81)	42 (78)	311 (82)
	**Urban**	**Rural**	**Northeast**	**Southern**	**Midwestern**	**Western**	**Total**
	n=241	n=139	n=83	n=195	n=48	n=54	n=380
**Have you been diagnosed with the following medical conditions? No. (%)**							
Dementia	2 (1)	1 (1)	1 (1)	2 (1)	0 (0)	0 (0)	3 (1)
Cancer	24 (10)	13 (9)	8 (10)	19 (10)	5 (10)	5 (9)	37 (10)
Heart/Vascular	65 (27)	29 (21)	18 (22)	43 (22)	15 (31)	18 (33)	94 (25)
Lung	11 (5)	3 (2)	4 (5)	5 (3)	4 (8)	1 (2)	14 (4)
Diabetes	75 (31)	40 (29)	21 (25)	62 (32)	15 (31)	17 (31)	115 (30)
Kidney	8 (3)	5 (4)	0 (0)	8 (4)	2 (4)	3 (6)	13 (3)
Other	20 (8)	15 (11)	9 (11)	16 (8)	5 (10)	5 (9)	35 (9)
None	88 (37)	67 (48)	33 (40)	88 (45)	17 (35)	17 (31)	155 (41)
Autoimmune	10 (4)	3 (2)	6 (7)	4 (2)	1 (2)	2 (4)	13 (3)

[a] **Reported percentages presented as column percentages**

© 2020 Van Scoy LJ et al. *JAMA Network Open*.

eTable 4. ACP Behavior Rates by Site, Demographic, and Region

	No.[a]	Com-pleted New AD	Updated, Reread, Or Created New AD	Talked To Loved Ones	Talked To Clinician	Discussed Game	Reviewed Resources	Funeral Planning[c]	Financial or insurance Planning[c]	Other Behavior[c]	1+ ACP Behaviors	3+ ACP Behaviors
**By Site, No. (%)[b]**												
Livingston, AL	28	13 (46)	14 (50)	23 (82)	7 (25)	20 (71)	17 (61)	1 (4)	2 (7)	1 (4)	28 (100)	18 (64)
Palo Alto, CA	14	4 (29)	5 (36)	7 (50)	3 (21)	10 (71)	9 (64)	2 (14)	3 (21)	1 (7)	13 (93)	7 (50)
Washington, DC	30	9 (30)	14 (47)	20 (67)	1 (3)	21 (70)	15 (50)	2 (7)	1 (3)	3 (10)[f]	29 (97)	17 (57)[e]
Lakeland, FL	14	5 (36)	5 (36)	12 (86)	0 (0)	13 (93)	8 (57)	0 (0)	3 (21)	1 (7)	14 (100)	11 (79)
Atlanta, GA	19	9 (47)	10 (53)	13 (68)	4 (21)	11 (58)	12 (63)	1 (5)	4 (21)	1 (5)	17 (89)	14 (74)
Chicago, IL	8	2 (25)	4 (50)	7 (88)	3 (38)	3 (38)	5 (63)	0 (0)	0 (0)	1 (13)[d]	8 (100)	5 (63)[d]
Broussard, LA	26	12 (46)	13 (50)	24 (92)	5 (19)	19 (73)	15 (58)	3 (12)	2 (8)	1 (4)[d]	26 (100)	18 (69)
St. Louis, MO	11	3 (27)	4 (36)	10 (91)	5 (45)	6 (55)	8 (73)	0 (0)	1 (9)	1 (9)[d]	11 (100)	8 (73)
Asheville, NC	12	7 (58)	8 (67)	9 (75)	0 (0)	10 (83)	6 (50)	1 (8)	0 (0)	0 (0)	11 (92)	9 (75)[d]
Las Vegas, NV	20	8 (40)	8 (40)	18 (90)	4 (20)	14 (70)	7 (35)	2 (10)	2 (10)	0 (0)	20 (100)	12 (60)
Sodus, NY	9	5 (56)	6 (67)	8 (89)	3 (33)	7 (78)	7 (78)	0 (0)	0 (0)	0 (0)	9 (100)	7 (78)
Philadelphia, PA	10	9 (90)	9 (90)	8 (80)	3 (30)	7 (70)	7 (70)[d]	1 (10)	2 (20)	0 (0)	9 (90)	8 (80)
Amarillo, TX	8	3 (38)	3 (38)	7 (88)	1 (13)	6 (75)	2 (25)	0 (0)	0 (0)	2 (25)	8 (100)	4 (50)
Milwaukee, WI	11	2 (18)	3 (27)	10 (91)	4 (36)	7 (63)	4 (36)	1 (9)	0 (0)	2 (18)[d]	11 (100)	7 (63)
**By Demographics, No. (%)**												
Urban sites	145	54 (37)	65 (45)	112 (77)	28 (19)	98 (68)	77 (53)[d]	9 (6)	16 (11)[h]	12 (8)[h]	140 (97)	93 (64)[f]
Rural sites	75	37 (49)	41 (55)	64 (85)	15 (20)	56 (75)	45 (60)	5 (7)	4 (5)[e]	2 (3)[e]	74 (99)[d]	52 (69)[d]
**By Region, No. (%)**												
Northern sites	49	23 (47)	29 (59)	36 (73)	7 (14)	35 (71)	29 (59)[d]	3 (6)	3 (6)[f]	3 (6)[f]	47 (96)	32 (65)[e]
Southern sites	107	49 (46)	53 (50)	88 (82)	17 (16)	79 (74)	60 (56)	6 (6)	11 (10)[e]	6 (6)[e]	104 (97)[c]	74 (69)[d]
Midwestern sites	30	7 (23)	11 (37)	27 (90)	12 (40)	16 (53)	17 (57)	1 (3)	1 (3)[e]	4 (13)[e]	30 (100)	20 (67)[e]
Western sites	34	12 (35)	13 (38)	25 (74)	7 (21)	24 (71)	16 (47)	4 (12)	5 (15)	1 (3)	33 (97)	19 (56)
**Total Follow-up Sample, No. (%)**	**220**	**91 (41)**	**106 (48)**	**176 (80)**	**43 (20)**	**154 (70)**	**122 (56)**[d]	**14 (6)**	**20 (9)**[i]	**14 (6)**[i]	**214 (98)**[d]	**145 (67)**[g]

[a] No. of Participants
[b] Reported percentages reported as row percentages
[c] Categories emerged from open-ended behavior prompt
[d]Missing n=1; [e]*Missing n=2;*[f]*Missing n=4;* [g]*Missing n=5;* [h]Missing n=*6;* [i]*Missing n=8*

© 2020 Van Scoy LJ et al. *JAMA Network Open*.

eTable 5. Mixed-Methods Joint Display

Quantitative data	Qualitative data[a]	
	Subtheme	Quotes, site (participant No.)
**Construct 1: Satisfaction with game experience**		
Satisfaction measures	Theme 1: The game was a useful forum for ACP conversations	
Conversation satisfaction	1A. Fun experience, enjoyed the game and being with community	… I found that, very refreshingly with the game, it was presented in a context that was quite non-threatening and in fact even, you know, kind of jovial at times … It was something, you know, that was kind of inviting enough that it drew you in
Mean (SD): 6.21 (0.93) [1 = low, 7 = high]		
Range of mean scores of sites: 5.58-6.88		
Conversation realism	1B. Safe space to discuss issues	It did a great job taking a very difficult topic and making it a little more lighthearted… Putting it in a game format lightened the heaviness of the topic. even though we had some discussions and we shed some tears….
	Theme 2: Participants gained new informative and perspective from the game experience	
	2A. Gained new context that improved understanding of ACP	It was very informative. It helped me to make definite decisions on if anything should happen to me … if they need to 'do not resuscitate' or let life continue with me to see. It just made me think about those situations and the quality of life depending on the issue that could occur in my life journey
	2B. Learned from perspectives of others	I enjoyed the fact that you actually got to hear someone else's view on past, life expectancy, or what they felt was important in their life. The sharing I think helps others so you don't think, 'Oh, I'm the only one who was thinking like this'
	Theme 3: Game experience was emotionally beneficial to the participants	
	3A. Provided comfort or relief for anxiety and fear about end-of-life	…If you have something inside of you and you keep it bottled up it just makes things worse. But once you get it out and start talking about it…you feel better and you've got it out in the open
	3B. Received positive reinforcement for having already completed ACP	It makes me look at my days a little bit better. 'Cause sometimes you be afraid of different things, but right now I'm really not afraid of anything 'cause I realize that I have everything in order … to know that my stuff is situated…that's what gave me the peace of mind that I needed
	3C. Felt fulfillment from sharing with and teaching others about ACP	it gave me a chance to help out other people, and I think it meet me in there being always prepared that showed them that they could do it too … it's not such a hard thing
Mean (SD): 5.20 (1.01) [1 = low, 7 = high]		
Range of mean scores of sites: 4.95-5.40		

© 2020 Van Scoy LJ et al. *JAMA Network Open*.

**Construct 2: Acceptability and recommendation**		
Measures	Theme 4: The game increased appreciation for both the need for and value of ACP	
Raw NPS	4A. Raised awareness about importance of ACP	it's made me aware of what I hadn't planned…you always think about the planning for the funeral, you don't think about planning for the health issues or hospitalization ...
Mean (SD): 8.76 (2.02) [0 = low endorsement, 10 = high endorsement]		
Range of mean scores of sites: 7.21 to 9.75		
Calculated NPS	4B. Emphasized urgency of planning for end-of-life issues	It was an eye-opener and a wake-up call that you need to get your affairs in order. You never know when that day is coming. You should go ahead and make your decisions on what you want …
Overall score: 57.89 (−100 = low endorsement, 100 = high endorsement)		
Range of scores of sites: 5.88 to 90.91		
**Construct 3: Self-efficacy and motivation for behavioral change**		
Behavioral measures	Theme 5: The game empowered and motivated participants to perform ACP	
Behavioral rates	NA	It has promoted me to go on and put some things in action where I hadn't. I was aware of a lot of the things they talked about, but I had never acted upon certain aspects of it. Thinking about tomorrow, something could expectantly happen, so it prompted me to try to act sooner on it
41% completed new AD		
48% updated, reread, or created new AD		
80% talked to loved ones		
98% completed ≥1 ACP behavior		
67% completed ≥3 ACP behaviors		
ACP-E-34, mean (SD)	NA	
Change in knowledge: 0.38 (1.24)		
Change in contemplation: 0.12 (1.12)		
Change in self-efficacy: 0.54 (0.98)		
Change in readiness: 0.33 (0.98)		
Change in total score: 0.40 (0.74)		

Abbreviations: ACP, advance care planning; ACP-E-34, ACP Engagement Survey 34-Item; AD, advance directive; NA, not applicable; NPS, Net Promoter Score.

[a]Additional qualitative quotations can be found in eTable 7 in the Supplement.

© 2020 Van Scoy LJ et al. *JAMA Network Open*.

eTable 6. ACP Engagement Survey Results by Site, Demographic, and Region

Knowledge Domain	Participants, No.	Post/Pre Paired n	Pre-Game Mean (SD)	Follow-Up Mean (SD)	Change Post – Pre (SD)	*P* Value
**By Site**						
Livingston, AL	28	28	3.89 (1.17)	3.52 (1.21)	-0.38 (1.28)	
Palo Alto, CA	14	13	3.62 (0.98)	4.12 (0.96)	+0.50 (1.21)	
Washington, DC	30	30	3.17 (1.23)	3.93 (0.73)	+0.77 (1.12)	
Lakeland, FL	14	14	3.57 (1.17)	4.11 (0.63)	+0.54 (1.26)	
Atlanta, GA	19	18	3.81 (1.06)	4.36 (0.82)	+0.56 (1.16)	
Chicago, IL	8	6	4.50 (0.84)	3.92 (1.56)	-0.58 (1.74)	
Broussard, LA	26	24	3.65 (1.26)	4.35 (0.77)	+0.71(1.01)	
St. Louis, MO	11	11	4.09 (1.22)	4.27 (1.01)	+0.18 (1.38)	
Asheville, NC	12	11	3.41 (1.02)	3.77 (0.85)	+0.36 (0.74)	
Las Vegas, NV	20	20	3.33 (1.07)	3.78 (1.02)	+0.45 (1.34)	
Sodus, NY	9	8	3.94 (1.15)	3.69 (1.25)	-0.25 (1.49)	
Philadelphia, PA	10	9	3.56 (1.13)	4.39 (0.65)	+0.83 (0.75)	
Amarillo, TX	8	8	3.75 (1.07)	3.94 (0.82)	+0.19 (1.00)	
Milwaukee, WI	11	10	3.50 (1.18)	4.35 (0.94)	+0.85 (1.29)	
**By Demographic**						
Urban Sites	145	139	3.57 (1.14)	4.09 (0.88)	+0.52 (1.22)	<.001
Rural Sites	75	71	3.74 (1.17)	3.86 (1.08)	+0.12 (1.23)	0.26
**By Region**						
Northeastern sites	49	47	3.37 (1.21)	3.98 (0.83)	+0.61 (1.17)	<.001
Southern sites	107	103	3.71 (1.13)	4.00 (0.96)	+0.29 (1.18)	0.009
Midwestern sites	30	27	3.96 (1.16)	4.22 (1.09)	+0.26 (1.48)	0.30
Western sites	34	33	3.44 (1.03)	3.91 (1.00)	+0.47 (1.27)	0.04
**Total Follow-up Sample**	**220**	**210**	**3.63 (1.15)**	**4.01 (0.96)**	**+0.38 (1.24)**	**<.001**
**Contemplation Domain**	**Participants, No.**	**Post/Pre Paired n**	**Pre-Game Mean (SD)**	**Follow-up Mean (SD)**	**Change Post – Pre (SD)**	***P* Value**
**By Site**						
Livingston, AL	28	27	3.19 (1.15)	3.38 (1.09)	+0.20 (1.13)	
Palo Alto, CA	14	13	3.05 (1.27)	3.44 (1.23)	+0.38 (0.89)	
Washington, DC	30	28	3.10 (1.17)	3.29 (1.10)	+0.19 (0.99)	
Lakeland, FL	14	13	3.21 (1.11)	3.05 (0.89)	-0.15 (1.37)	
Atlanta, GA	19	16	2.94 (1.13)	3.08 (0.91)	+0.15 (0.90)	
Chicago, IL	8	6	4.44 (0.50)	3.67 (0.94)	-0.78 (0.98)	
Broussard, LA	26	24	2.90 (1.08)	3.31 (0.85)	+0.40 (1.16)	

© 2020 Van Scoy LJ et al. *JAMA Network Open*.

Contemplation Domain (continued)	Participants, No.	Post/Pre Paired n	Pre-Game Mean (SD)	Follow-up Mean (SD)	Change Post – Pre (SD)	*P* Value
St. Louis, MO	11	8	4.00 (1.04)	3.63 (1.17)	-0.38 (0.90)	
Asheville, NC	12	10	3.00 (1.35)	3.47 (1.01)	+0.47 (1.21)	
Las Vegas, NV	20	17	3.35 (1.12)	3.22 (0.80)	-0.14 (1.34)	
Sodus, NY	9	7	3.43 (0.83)	3.33 (1.15)	-0.10 (0.94)	
Philadelphia, PA	10	9	3.26 (1.09)	3.70 (1.35)	+0.44 (1.39)	
Amarillo, TX	8	8	3.88 (0.96)	3.38 (0.63)	-0.50 (0.99)	
Milwaukee, WI	11	10	3.47 (0.95)	3.90 (0.42)	+0.43 (1.16)	
**By Demographic**						
Urban Sites	145	128	3.33 (1.13)	3.36 (0.99)	+0.04 (1.12)	0.58
Rural Sites	75	68	3.08 (1.12)	3.36 (0.98)	+0.28 (1.12)	0.08
**By Region**						
Northeastern sites	49	44	3.18 (1.09)	3.38 (1.15)	+0.20 (1.06)	0.22
Southern sites	107	98	3.12 (1.13)	3.28 (0.93)	+0.16 (1.14)	0.24
Midwestern sites	30	24	3.89 (0.95)	3.75 (0.84)	-0.14 (1.12)	0.56
Western sites	34	30	3.22 (1.18)	3.31 (0.99)	+0.09 (1.18)	0.71
**Total Follow-up Sample**	**220**	**196**	**3.24 (1.13)**	**3.36 (0.98)**	**+0.12 (1.12)**	**0.16**
**Self-Efficacy Domain**	**Participants, No.**	**Post/Pre Paired n**	**Pre-Game Mean (SD)**	**Follow-up Mean (SD)**	**Change Post – Pre (SD)**	***P* Value**
**By Site**						
Livingston, AL	28	23	3.38 (1.03)	4.05 (1.16)	+0.67 (1.11)	
Palo Alto, CA	14	9	3.03 (1.29)	3.98 (1.02)	+0.95 (0.66)	
Washington, DC	30	24	3.61 (0.84)	4.07 (0.84)	+0.45 (0.86)	
Lakeland, FL	14	11	3.53 (1.06)	4.12 (0.87)	+0.59 (1.18)	
Atlanta, GA	19	14	3.39 (1.19)	4.00 (0.92)	+0.61 (1.30)	
Chicago, IL	8	6	4.32 (0.68)	4.50 (0.70)	+0.18 (0.39)	
Broussard, LA	26	24	3.48 (1.16)	4.29 (0.45)	+0.81 (0.99)	
St. Louis, MO	11	9	4.07 (0.64)	4.30 (0.71)	+0.22 (0.56)	
Asheville, NC	12	9	3.70 (1.09)	4.15 (0.72)	+0.44 (0.51)	
Las Vegas, NV	20	11	3.90 (0.64)	3.92 (0.88)	+0.02 (1.04)	
Sodus, NY	9	7	3.85 (0.91)	4.55 (0.58)	+0.70 (0.99)	
Philadelphia, PA	10	9	3.83 (1.17)	4.49 (0.57)	+0.66 (1.24)	
Amarillo, TX	8	7	4.13 (1.10)	4.37 (0.52)	+0.24 (1.01)	
Milwaukee, WI	11	10	3.88 (0.70)	4.31 (0.68)	+0.43 (1.01)	
**By Demographic**						
Urban Sites	145	110	3.71 (0.97)	4.16 (0.80)	+0.45 (0.98)	<.001
Rural Sites	75	63	3.52 (1.06)	4.21 (0.82)	+0.70 (0.97)	<.001

© 2020 Van Scoy LJ et al. *JAMA Network Open*.

Self-Efficacy Domain (continued)	Participants, No.	Post/Pre Paired n	Pre-Game Mean (SD)	Follow-up Mean (SD)	Change Post – Pre (SD)	*P* Value
**By Region**						
Northeastern sites	49	40	3.70 (0.91)	4.25 (0.76)	+0.54 (0.96)	<.001
Southern sites	107	88	3.52 (1.09)	4.15 (0.83)	+0.63 (1.05)	<.001
Midwestern sites	30	25	4.05 (0.67)	4.35 (0.67)	+0.30 (0.73)	0.05
Western sites	34	20	3.51 (1.05)	3.95 (0.92)	+0.44 (0.99)	0.06
**Total Follow-up Sample**	**220**	**173**	**3.64 (1.01)**	**4.18 (0.81)**	**+0.54 (0.98)**	**<.001**
**Readiness Domain**	**Participants, No.**	**Post/Pre Paired n**	**Pre-Game Mean (SD)**	**Follow-up Mean (SD)**	**Change Post – Pre (SD)**	***P* Value**
**By Site**						
Livingston, AL	28	21	2.62 (1.33)	2.83 (0.76)	+0.21 (1.30)	
Palo Alto, CA	14	10	2.73 (1.30)	2.71 (1.28)	-0.02 (0.52)	
Washington, DC	30	22	2.70 (0.91)	3.16 (0.91)	+0.46 (0.79)	
Lakeland, FL	14	12	2.90 (1.12)	3.11 (0.65)	+0.21 (0.83)	
Atlanta, GA	19	11	2.28 (0.76)	3.06 (1.11)	+0.78 (1.00)	
Chicago, IL	8	4	4.26 (0.53)	3.74 (1.41)	-0.53 (1.76)	
Broussard, LA	26	22	2.48 (0.97)	3.08 (0.78)	+0.60 (0.98)	
St. Louis, MO	11	7	3.51 (0.96)	3.68 (0.80)	+0.17 (0.79)	
Asheville, NC	12	10	3.16 (0.96)	3.51 (0.87)	+0.34 (0.76)	
Las Vegas, NV	20	15	2.90 (1.02)	3.31 (0.66)	+0.42 (0.80)	
Sodus, NY	9	5	3.07 (1.10)	3.66 (0.92)	+0.59 (0.85)	
Philadelphia, PA	10	8	3.04 (1.36)	2.96 (1.22)	-0.08 (1.57)	
Amarillo, TX	8	8	3.38 (1.06)	3.49 (0.80)	+0.10 (1.06)	
Milwaukee, WI	11	9	3.29 (0.87)	3.71 (0.68)	+0.42 (0.74)	
**By Demographic**						
Urban sites	145	106	2.95 (1.06)	3.23 (0.94)	+0.28 (0.94)	<.001
Rural sites	75	58	2.70 (1.13)	3.11 (0.83)	+0.41 (1.06)	.01
**By Region**						
Northeastern sites	49	35	2.83 (1.03)	3.18 (0.98)	+0.35 (1.02)	.02
Southern sites	107	84	2.72 (1.10)	3.11 (0.83)	+0.39 (1.03)	<.001
Midwestern sites	30	20	3.56 (0.89)	3.71 (0.84)	+0.14 (1.02)	0.20
Western sites	34	25	2.83 (1.12)	3.07 (0.98)	+0.24 (0.73)	0.13
**Total Follow-up Sample**	**220**	**164**	**2.86 (1.09)**	**3.19 (0.90)**	**+0.33 (0.98)**	**<.001**
**34-Item Total Score**	**Participants, No.**	**Post/Pre Paired n**	**Pre-Game Mean (SD)**	**Follow-up Mean (SD)**	**Change Post – Pre (SD)**	***P* Value**
**By Site**						
Livingston, AL	28	18	2.96 (0.90)	3.42 (0.72)	+0.45 (0.92)	
Palo Alto, CA	14	8	2.97 (1.33)	3.40 (1.11)	+0.42 (0.29)	
Washington, DC	30	21	3.07 (0.88)	3.49 (0.80)	+0.43 (0.73)	
Lakeland, FL	14	10	3.15 (1.00)	3.52 (0.68)	+0.37 (0.76)	
Atlanta, GA	19	10	2.82 (0.86)	3.31 (0.85)	+0.49 (0.76)	

© 2020 Van Scoy LJ et al. *JAMA Network Open*.

34-Item Total Score (continued)	Participants, No.	Post/Pre Paired n	Pre-Game Mean (SD)	Follow-up Mean (SD)	Change Post – Pre (SD)	*P* Value
Chicago, IL	8	2	3.90 (0.40)	4.44 (0.46)	+0.54 (0.06)	
Broussard, LA	26	20	2.87 (0.92)	3.54 (0.52)	+0.67 (0.76)	
St. Louis, MO	11	7	3.73 (0.82)	3.84 (0.70)	+0.11 (0.67)	
Asheville, NC	12	9	3.37 (0.97)	3.71 (0.72)	+0.34 (0.51)	
Las Vegas, NV	20	11	3.34 (0.64)	3.55 (0.66)	+0.21 (0.62)	
Sodus, NY	9	5	3.56 (0.54)	4.00 (0.78)	+0.44 (0.44)	
Philadelphia, PA	10	8	3.32 (1.13)	3.61 (0.84)	+0.29 (1.21)	
Amarillo, TX	8	7	3.71 (1.00)	3.88 (0.62)	+0.17 (0.90)	
Milwaukee, WI	11	8	3.61 (0.53)	3.95 (0.49)	+0.34 (0.69)	
**By Demographic**						
Urban sites	145	92	3.26 (0.92)	3.60 (0.76)	+0.34 (0.73)	<.001
Rural sites	75	52	3.05 (0.91)	3.57 (0.66)	+0.52 (0.75)	<.001
**By Region**						
Northeastern sites	49	34	3.20 (0.90)	3.59 (0.80)	+0.40 (0.81)	0.01
Southern sites	107	74	3.06 (0.94)	3.53 (0.67)	+0.47 (0.78)	<.001
Midwestern sites	30	17	3.69 (0.63)	3.96 (0.58)	+0.27 (0.64)	.10
Western sites	34	19	3.19 (0.98)	3.48 (0.86)	+0.30 (0.51)	0.02
**Total Follow-up Sample**	**220**	**144**	**3.19 (0.92)**	**3.59 (0.73)**	**+0.40 (0.74)**	**<.001**
**4-Item Total Score^**	**Participants, No.**	**Post/Pre Paired n**	**Pre-Game Mean (SD)**	**Follow-up Mean (SD)**	**Change Post – Pre (SD)**	***P* Value**
**By Site**						
Livingston, AL	28	27	2.57 (1.40)	3.22 (0.90)	+0.65 (1.32)	
Palo Alto, CA	14	12	3.08 (1.51)	2.81 (1.64)	-0.27 (0.58)	
Washington, DC	30	26	2.88 (0.90)	3.12 (0.83)	+0.24 (0.91)	
Lakeland, FL	14	12	2.79 (1.06)	3.35 (0.86)	+0.56 (0.98)	
Atlanta, GA	19	16	2.30 (0.61)	2.81 (0.87)	+0.52 (0.90)	
Chicago, IL	8	5	4.25 (0.35)	3.80 (1.15)	-0.45 (1.35)	
Broussard, LA	26	26	2.47 (1.08)	3.20 (0.89)	+0.73 (0.98)	
St. Louis, MO	11	9	3.64 (1.15)	4.03 (1.11)	+0.39 (0.67)	
Asheville, NC	12	10	3.30 (0.99)	3.50 (0.96)	+0.20 (0.71)	
Las Vegas, NV	20	19	3.07 (1.05)	3.41 (0.79)	+0.34 (0.91)	
Sodus, NY	9	7	3.50 (1.31)	3.64 (0.85)	+0.14 (1.10)	
Philadelphia, PA	10	9	3.53 (1.54)	3.22 (1.26)	-0.31 (1.49)	
Amarillo, TX	8	8	3.41 (1.05)	3.41 (0.84)	0.00 (0.93)	
Milwaukee, WI	11	9	3.50 (0.88)	3.75 (0.67)	+0.25 (0.88)	
**By Demographic**						
Urban sites	145	125	3.08 (1.11)	3.28 (1.02)	+0.20 (0.96)	.01
Rural sites	75	70	2.73 (1.26)	3.30 (0.90)	+0.56 (1.11)	<.001
**By Region**						
Northeastern sites	49	42	3.12 (1.15)	3.23 (0.94)	+0.11 (1.08)	.36

© 2020 Van Scoy LJ et al. *JAMA Network Open*.

4-Item Total Score (continued)	Participants, No.	Post/Pre Paired n	Pre-Game Mean (SD)	Follow-up Mean (SD)	Change Post – Pre (SD)	*P* Value
Southern sites	107	99	2.67 (1.14)	3.21 (0.89)	+0.54 (1.05)	<.001
Midwestern sites	30	23	3.72 (0.93)	3.87 (0.93)	+0.15 (0.94)	.32
Western sites	34	31	3.07 (1.22)	3.18 (1.20)	+0.10 (0.84)	0.61
**Total Follow-up Sample**	**220**	**195**	**2.95 (1.17)**	**3.29 (0.98)**	**+0.33 (1.03)**	**<.001**

© 2020 Van Scoy LJ et al. *JAMA Network Open*.

eTable 7. Net Promoter Score, Conversation Satisfaction, and Conversation Realism Results by Site, Demographic, and Region

	**Participants, No.**	**Conversation Satisfaction** Mean (SD) 1=Lowest satisfaction 7=Highest satisfaction	**Missing No.**	**Conversation Realism** Mean (SD) 1=Not at all realistic 7=Very realistic	**Missing No.**	**Net Promoter Score (Raw)** Mean (SD) 1=Not at all likely to recommend 10=Extremely likely to recommend	**Missing No.**	**Net Promoter Score (Calculated)** Difference in percentages Range -100 to +100
**By Site**								
Livingston, AL	50	5.93 (0.92)	2	5.40 (1.54)	3	8.98 (1.94)	4	60.87
Palo Alto, CA	18	6.08 (1.03)	0	5.04 (1.00)	1	8.39 (2.23)	0	44.45
Washington, DC	45	6.34 (0.79)	5	5.27 (0.76)	6	8.83 (1.69)	5	57.50
Lakeland, FL	23	6.55 (0.57)	1	5.12 (1.01)	3	9.59 (0.80)	1	90.91
Atlanta, GA	35	5.97 (1.04)	2	5.12 (0.82)	3	7.21 (3.11)	1	5.88
Chicago, IL	13	6.04 (1.02)	1	5.05 (0.70)	2	7.38 (3.07)	0	23.08
Broussard, LA	43	6.68 (0.56)	1	5.38 (0.65)	2	9.40 (1.04)	1	78.57
St. Louis, MO	17	6.88 (0.32)	1	5.05 (0.75)	1	9.75 (0.68)	1	87.50
Asheville, NC	24	6.04 (1.15)	1	5.14 (0.89)	3	8.86 (2.12)	2	68.18
Las Vegas, NV	36	5.91 (1.11)	2	5.23 (1.22)	5	8.79 (1.68)	2	55.88
Sodus, NY	22	5.58 (0.98)	2	4.86 (1.27)	0	8.29 (2.03)	1	42.85
Philadelphia, PA	16	6.22 (1.06)	2	5.40 (0.76)	1	8.69 (2.30)	0	68.75
Amarillo, TX	20	6.74 (0.51)	3	5.27 (0.57)	1	9.37 (1.12)	1	73.68
Milwaukee, WI	18	6.24 (0.83)	0	4.95 (1.02)	1	8.78 (1.90)	0	55.55
**By Demographics**								
Urban sites	241	6.26 (0.92)	17	5.17 (0.89)	24	8.63 (2.14)	11	53.92
Rural sites	139	6.13 (0.96)	6	5.26 (1.18)	8	8.98 (1.77)	8	64.89
**By Region**								
Northeastern sites	83	6.11 (0.94)	9	5.18 (0.94)	7	8.65 (1.91)	6	55.84
Southern sites	195	6.27 (0.90)	10	5.27 (1.03)	15	8.85 (2.07)	10	60.54
Midwestern sites	48	6.41 (0.82)	2	5.01 (0.83)	4	8.72 (2.19)	1	57.45
Western sites	54	5.97 (1.08)	2	5.16 (1.14)	6	8.65 (1.88)	2	51.93
**Total Sampling**	380	6.21 (0.93)	23	5.20 (1.01)	32	8.76 (2.02)	19	57.89

© 2020 Van Scoy LJ et al. *JAMA Network Open*.

eTable 8. Additional Qualitative Quotations from Phone Interviews	
**Theme 1: The game was an effective forum for ACP conversations**	
**Subtheme**	**Quotes**
1A) Fun experience, enjoyed the game and being with community	… It lifted you up a little, and I found a lot of joy, it wasn't sad. Even in discussing, it was funny in a lot of things we were talking about …"
2A) Safe space to discuss issues	I enjoyed the open environment– people could talk and express themselves without judgment or feeling guilty about having the conversation. It was comfortable because you're not alone.<br>I think it was a less intimidating way to talk about issues that most people avoid.<br>I felt like playing the Hello Game made me open up more in that environment rather than thinking about it by myself or with my spouse. It was easier to communicate
**Theme 2: Participants gained new informative and perspective from the game experience**	
**Subtheme**	**Quotes**
2A) Gained new context that improved understanding of ACP	It has made me more aware that I do need to make plans before my exit from this planet. All I have is insurance. I haven't explained to anyone what I would like in certain situations.<br>It brought out a lot of information that will clear up some things that I was afraid of. It cleared up a lot of things and helped me understand better."<br>Well, it gave me sort of an introduction into what things are necessary to be done and how I could go about doing it, and so on. And to try to look at the – the situation you're in and see how – how things could work out in that situation."
2B) Learned from perspectives of others	It gave us an opportunity to listen to other people talk about how they feel about end-of-life issues and how they handle them. That sharing gave me a lot more thought on how I look at things and it's not just about me, but everyone else and how they are affected by my passing. It helped me grow up a bit.<br>It allowed me to get to know some of my fellow church members. We were a variety of age groups at different places and stages of our lives. It was nice to have an open forum where we could talk honestly about end life care. What we found out is that it might be easier to tell a close friend rather than a child or a parent what your end of life wishes are.<br>I thought it was really – it was amazing. I mean, some of the people, what they did and how they cope with certain things … I really enjoyed the people at the table. They were very informative and willing to give you some advice…<br>Listening to the others helps you think a little more about where you are and where you would like to be and are you prepared for [what] is to come at a certain point."<br>… I appreciated that fact that I got to hear not just my perspective … but other people's perspective on the same question, because sometimes it's so easy to get stuck in just the way you think about advance care planning or, you know, issues related to death and dying …"
**Theme 3: Game experience was emotionally beneficial to the participants**	
**Subtheme**	**Quote**

© 2020 Van Scoy LJ et al. *JAMA Network Open*.

3A) Provided comfort or relief for anxiety and fear about end-of-life	... it made me think more of what I would want at the time of death and how to live my life now. And to let go of things that really was holding me back or just making me angry …"
3B) Received positive reinforcement for having already completed ACP	I made my arrangements, my brother made his, I still have some siblings who haven't…it was comforting to know that it had been taken care of…for the most part and it wasn't unfamiliar.<br>I thought more about my decision that I had made because it's good to have a will in place…and my niece is my power of attorney.<br>I had already taken care of my business before then, but I did get one of my friends to take care of her business.
3C) Felt fulfillment from sharing with and teaching others about ACP	I've never feared death … So, to be able to tell my story to others, and at the end I found out it had made a lot of people more comfortable …"
**Theme 4: The game increased appreciation for both the need for and value of ACP**	
**Subtheme**	**Quote**
4A) Raised awareness about importance of ACP	Playing the Hello game got me thinking about the importance of having these conversations and discussions and if you don't do it ahead of time, when you need it, it's too late…it was an approach to initiating and pretty much controlling the conversation, you being in control<br>It was an eye-opener, because it hasn't crossed my mind. I've had some of my loved ones pass away—my dad, my brother, my sister…But [the game] was like an eye-opener.<br>It made me more aware of things that I took for granted. Like planning your affairs or if something happens to me and I can't take care of myself anymore. It let me know there's things you can do and people you can call and get the help you need or help your family.
4B) Emphasized urgency of planning for end-of-life issues	It made you realize that this is something that you shouldn't procrastinate about, it needs to be be done. It is important to make sure that there is no unhappiness when you should have peace. It is something we should plan for just like everything else in our lives.<br>It made me know that I need to do something soon. Because I had planned on doing a will and what-not, but not a lot of the other things, issues I hadn't really thought about …"
**Theme 5: The game empowered and motivated participants to perform ACP**	
**Subtheme**	**Quote**
N/A	It just made me ready to give me the nerve to go and be able to do things—talk to my doctor and my lawyer<br>I was able to discuss things. It gave me ideas about how and what I needed to talk to my family about. Again, it opened up avenues that I probably wouldn't have taken and initiated the conversation with my family.

© 2020 Van Scoy LJ et al. *JAMA Network Open*.
